# Effect of the combined addition of ultrasonicated kraft lignin and montmorillonite on hydroxypropyl methylcellulose bionanocomposites†

**DOI:** 10.1039/d3na00283g

**Published:** 2023-07-10

**Authors:** Raquel Martín-Sampedro, Pilar Aranda, Gustavo del Real, Eduardo Ruiz-Hitzky, Margarita Darder

**Affiliations:** a Materials Science Institute of Madrid (ICMM), CSIC C/ Sor Juana Inés de la Cruz 3 28049 Madrid Spain raquel.martin@inia.csic.es; b Institute of Forest Sciences (ICIFOR), INIA – CSIC Ctra. de la Coruña, km 7.5 28040 Madrid Spain; c Interdisciplinary Platform for Sustainable Plastics towards a Circular Economy-Spanish National Research Council (SusPlast-CSIC) Madrid Spain; d National Institute of Agricultural and Food Research and Technology (INIA), CSIC Ctra. de la Coruña, km 7.5 28040 Madrid Spain

## Abstract

Although hydroxypropyl methylcellulose (HPMC) has been proposed as renewable substitute for traditional plastic, its barrier and active properties need to be improved. Thus, the combination of an organic residue such as kraft lignin (0–10% w/w) and a natural clay such as montmorillonite (3% w/w) by application of ultrasound can significantly improve HPMC properties. This is most likely due to the close interaction between lignin and montmorillonite, which leads to delamination of the clay and improves its dispersion within the HPMC matrix. Specifically, the addition of kraft lignin to the bionanocomposite films provided them with UV-shielding, antioxidant capacity and antibacterial activity. The incorporation of 3% montmorillonite resulted in reductions of 65.8% and 11.4% in oxygen (OP) and water vapor permeabilities (WVP), respectively. Moreover, a reduction of 43.8% in WVP was achieved when both lignin (1%) and montmorillonite (3%) were incorporated, observing a synergistic effect. Thus, the HPMC bionanocomposite with 1% lignin and 3% montmorillonite, presented good thermal stability and mechanical strength with significantly improved gas barrier permeability, as well as UV-shielding (maintaining a good transparency), antioxidant and antibacterial activities.

## Abstract 2

Lignin caused montmorillonite exfoliation improving clay compatibility with HPMC, and conferred UV-shielding, antioxidant and antibacterial properties, while montmorillonite improved barrier properties.

## Graphical abstract

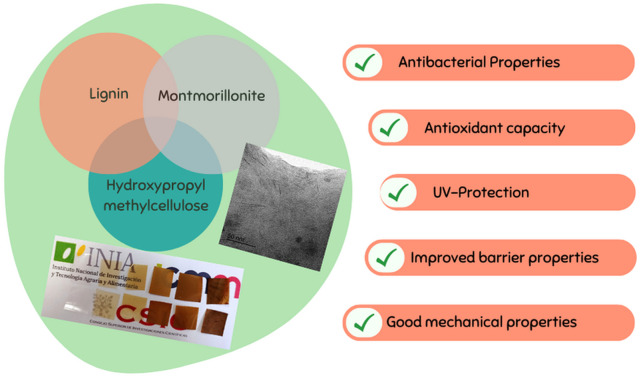

## 1. Introduction

One of the main concerns of today's society is the environmental problem associated with the extensive use of plastic, especially in packaging applications. These problems include not only the disposal of non-biodegradable plastic, the accumulation of microplastics or the emission of greenhouse gases, but also the dependence and use of fossil resources.^1,2^ Therefore, the replacement of traditional plastic by biodegradable bioplastic, obtained from renewable resources, will significantly contribute to the transition from a fossil fuel-based economy to a sustainable and circular bioeconomy. In this way, the search for new biodegradable, highly versatile, non-toxic and low-cost materials obtained from renewable resources, has attracted considerable interest in the last years, not only in Academia but also in the industrial field. Thus, the production capacities of these biodegradable plastics increase each year and are estimated to reach around 1.8 million tons in 2025.^2^

In the food packaging sector, an effective packaging should protect products from the external environment including mechanical forces, odors, dust, gases, moisture, radiation/light and microorganisms.^3^ Furthermore, packaging is also important to provide some information to customers and for marketing purposes.^3^ Adding to these requirements the need to use non-toxic, biodegradable and low-cost materials, several proteins, polysaccharides and lipid-based biopolymer materials have been proposed.^1,4,5^ Among polysaccharides, hydroxypropyl methylcellulose (HPMC) based-film has proved some advantages such as transparency, flexibility, odorless, tasteless, non-toxicity, edibility, good oxygen and grease barrier properties, and good film formability.^6–8^ However, like other polysaccharides, HPMC is water sensitive due to the presence of hydrophilic groups, showing poor moisture barrier properties. To overcome this limitation, different approaches have been proposed such as the incorporation of hydrophobic surfactants or lipid compounds^6–8^ or the addition of nanofillers such as clay-based materials.^9–13^ Among the different types of clays, montmorillonite is one of the most used in polymer nanocomposites, due to its low cost, high availability, relatively high cationic exchange capacity and easy expandability (allowing intercalation of different compounds). This clay is a 2 : 1 phyllosilicate belonging to the smectite group showing a structure consisting of layers of octahedral aluminium (partially substituted by magnesium) oxyhydroxide layers sandwiched between two layers of tetrahedral silica. Although montmorillonite has been incorporated as a nanofiller improving diverse properties of a large number of biopolymer matrices,^14,15^ only a few works are related with nanocomposites resulting from the addition of this clay to HPMC.^7,9,10,16^ These authors observed that not only barrier but also mechanical and thermal properties improved with the addition of this clay, as also observed in the case of other biopolymer nanocomposites.^14,15^

As mentioned above, food packaging should protect the products from the external environment, including light irradiation, microorganisms and food oxidation. Hence, active compounds from natural origin are often added to the bionanocomposites to improve food protection.^17,18^ Among them, lignin is a natural polymer that can endow the bionanocomposite with antioxidant and antimicrobial properties, UV-shielding and certain hydrophobicity due to its poly-aromatic structure and wide variety of functional groups.^2^ It is the second most abundant biopolymer on Earth, and it is usually obtained as residual fraction in most lignocellulose's transformation processes in pulp and paper industry and second generation ethanol production. Kraft pulping is the most extended process for the production of cellulose pulp, generating around 50–60 million tons of kraft lignin per year, which is normally burned to generate heat and electricity in the kraft mill.^1^ Only 2% of this lignin is isolated by acid precipitation and commercially used.^1^ The search for potential uses of this residual lignin will significantly contribute to the implementation of the lignocellulosic biorefineries and the transition to a circular bioeconomy. Due to its biocompatibility and nontoxicity, it can be applied in food packaging and biomedical materials.^2^ In this search, kraft lignin has been used as additive in different biopolymer nanocomposites based on starch, chitosan/chitin, gelatin, agar, alginate and soy protein among others,^2^ which showed an increase not only in UV-shielding, water stability, antimicrobial and antioxidant properties, but also in thermal and mechanical properties.^2^ However, as far as we know, the effect of lignin addition on HPMC nanocomposites has hardly been studied previously,^19^ and its combination with montmorillonite in HPMC nanocomposite has never been investigated.

Therefore, the objective of this work is to improve the properties of HPMC films by the addition of kraft lignin and montmorillonite. The effect of lignin has been studied varying the lignin content between 0 and 10%. However, the percentage of montmorillonite was fixed at 3%, based on previous works reported by Mondal *et al.*^10^ and Moura *et al.*^9^ In addition, ultrasound was applied to the individual components suspension and the lignin–montmorillonite mixture before addition to the HPMC solution. Thus, nanocomposite films of HPMC + lignin and HPMC + montmorillonite + lignin have been produced under ultrasonication and characterized for the first time. Mechanical, thermal, light and gas barrier, antibacterial and antioxidant properties of the resulting bionanocomposites were compared with those of pure HPMC film.

## 2. Materials and methods

### 2.1. Materials

Kraft lignin (L) was kindly provided by ENCE (Pontevedra, Spain). It was obtained by acid precipitation from black liquor originated during kraft cooking of *Eucalyptus globulus* and presented a purity of 98.2%. Montmorillonite (M) was supplied by Southern Clay Products and corresponds to the commercial product known as Cloisite®Na^+^. It is a Wyoming-type Na-montmorillonite with ideal formula Na_0.33_[(AlMg)_2_(Si_4_O_10_)(OH)_2_]·*n*H_2_O and cation exchange capacity (CEC) of 93 mEq/100 g.^16^ Hydroxypropyl methylcellulose (HPMC; ∼22 kDa; methoxyl content: 28–20%, hydroxypropoxyl content: 7–12%), 2,2′-azino-bis(3-ethylbenzthiazoline-6-sulphonic acid) diammonium salt (ABTS) and other chemicals used here were purchased from Sigma-Aldrich (Madrid, Spain), being used as received, *i.e.* without further treatments.

### 2.2. Bionanocomposite films preparation

**Fig. 1:**
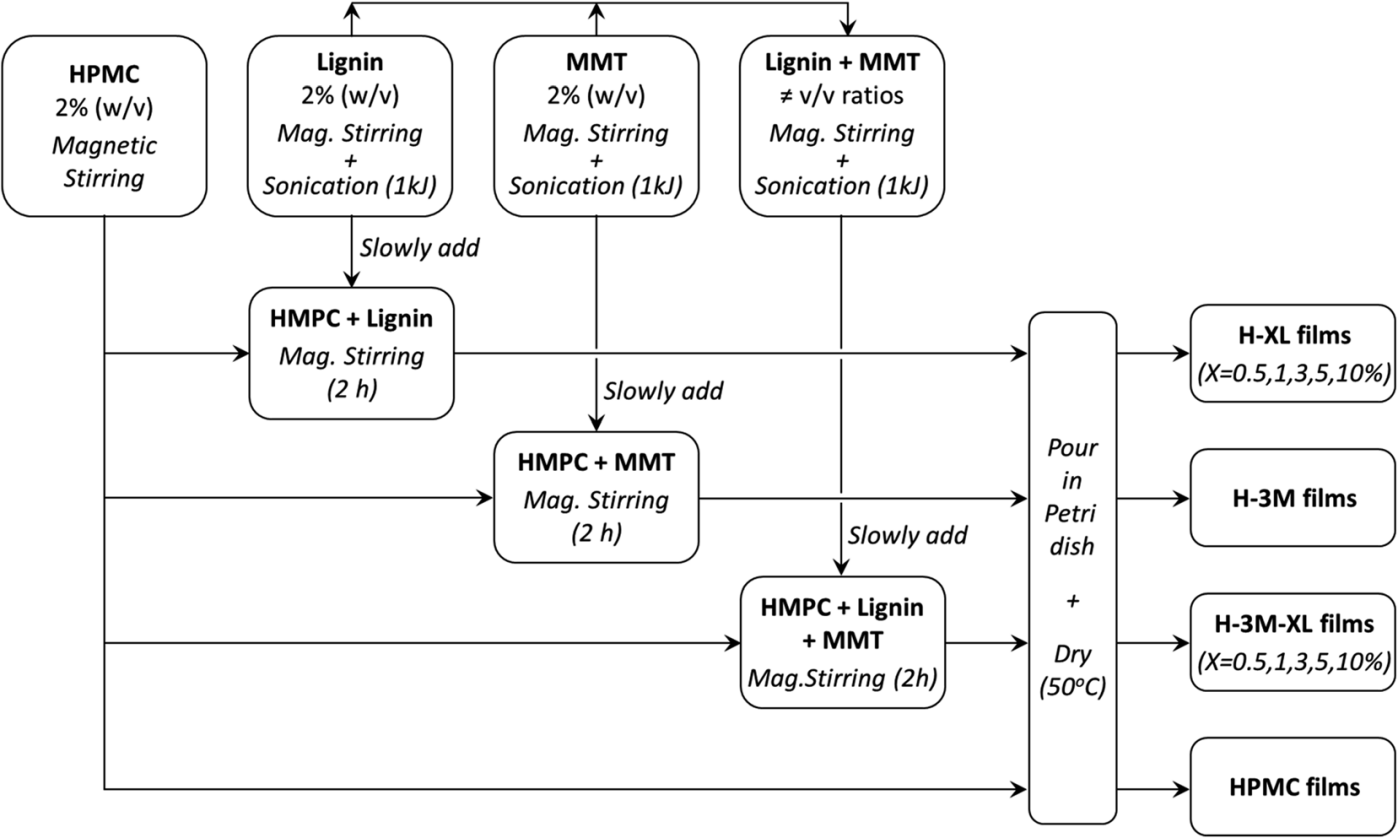
Flow diagram of the procedure followed for the preparation of the bionanocomposite films (MMT means montmorillonite).

HPMC films with variable lignin content (0.5%, 1.0%, 3.0%, 5.0% and 10% mass percentage with respect to the total mass of dry film) were prepared and labeled as H-*X*L, where *X* indicates the lignin content. Furthermore, to study the effect of the addition of a layered clay, similar films were prepared but adding a 3.0% (w/w) of montmorillonite being labeled as H-*X*L-3M. In addition, control samples of HPMC without lignin were prepared without or with 3% of montmorillonite: HPMC and H-3M, respectively. The procedure followed for the preparation of the bionanocomposite films was as follows: (1) 2% (w/v) HPMC solution was prepared by magnetic stirring using ultrapure water (Milli-Q); (2) solutions of 2% (w/v) lignin (in 0.5 M NaOH) and 2% (w/v) montmorillonite (in ultrapure water) were individually prepared by magnetic stirring for 30 minutes, followed by sonication (1 kJ total energy, 10 s on/off pulses and 50% amplitude) using a Vibra Cell VC750 ultrasonic processor equipped with a titanium sonication probe (13 mm diameter); (3) the 2% lignin solution and the 2% montmorillonite dispersion were mixed in the corresponding v/v ratio under magnetic stirring, followed by sonication at the same conditions described above (1 kJ total energy); (4) the desired volume of the lignin solution, montmorillonite dispersion or the lignin–montmorillonite mixture was slowly added to the HPMC solution under magnetic stirring, and left stirring for 2 hours to ensure complete homogenization; (5) 10 mL of each bionanocomposite mixture was poured in a Petri dish (54 mm diameter) and allowed to dry at 50 °C for several days. Table 1 shows the composition of the bionanocomposite films prepared. This procedure is summarized in Fig. 1.

**Table 1:** Composition of the bionanocomposite films: mass percentages of hydroxypropyl methylcellulose (HPMC), lignin and montmorillonite (MMT); and thickness and apparent density of the resulting films

Film	HPMC (w/w%)	Lignin (w/w%)	MMT (w/w%)	Thickness (μm)	Density (g cm^−3^)
H	100.0	0.0	0.0	51 ± 5	1.14 ± 0.12
H-0.5L	99.5	0.5	0.0	57 ± 4	1.07 ± 0.13
H-1L	99.0	1.0	0.0	50 ± 4	1.19 ± 0.10
H-3L	97.0	3.0	0.0	60 ± 5	1.08 ± 0.12
H-5L	95.0	5.0	0.0	56 ± 3	1.20 ± 0.06
H-10L	90.0	10.0	0.0	90 ± 7	0.89 ± 0.10
H-3M	97.0	0.0	3.0	52 ± 4	1.17 ± 0.12
H-3M-0.5L	96.5	0.5	3.0	56 ± 4	1.14 ± 0.11
H-3M-1L	96.0	1.0	3.0	55 ± 4	1.10 ± 0.08
H-3M-3L	94.0	3.0	3.0	53 ± 3	1.23 ± 0.06
H-3M-5L	92.0	5.0	3.0	62 ± 5	1.10 ± 0.08
H-3M-10L	87.0	10.0	3.0	92 ± 9	0.86 ± 0.08

### 2.3. Characterization of the bionanocomposite films

Field emission scanning electron microscopy (FE-SEM) was used to study the surface morphology of the bionanocomposite films, which were placed into a sticky carbon conductive tape and subjected to 30 seconds sputtering with Au. Images were acquired with a FEI-NOVA NanoSEM 230 microscope using a low voltage high contrast detector (vCD). Images of the films cross-section were also acquired using the same microscope with an Everhart–Thornley detector (ETD). To this end, samples were fractured after frozen with liquid nitrogen, and then sputtered with Au during 30 seconds.

In order to study the montmorillonite delamination in the bionanocomposites and the lignin–montmorillonite hybrids, a JEOL JEM-1400 Plus Transmission Electron Microscope (TEM) equipped with a Gatan ORIUS camera was used. Furthermore, lignin distribution in the film samples was evaluated by confocal microscopy, using a Zeiss LSM 800 microscope with highly sensitive GaAsP detectors. A laser diode at 488 nm (blue) was used as excitation source, emitting in green (561 nm).

X-ray powder diffraction was performed using a Bruker D8 Advance diffractometer (Bruker, USA) with CuKα radiation (Cu anode) and Ni filter, from 3° to 30° (2*θ*) with a step size of 0.04 and a goniometer speed of 0.5 second per step. Fourier-transform infrared (FTIR) spectra of the different films were directly acquired using the film samples in a Bruker iFS 66VS spectrometer (4000–400 cm^−1^ range, 400 scans, 1 cm^−1^ resolution). TG analyses were performed using a SDT-Q600 thermogravimetric analyzer (TA Instrument) under N_2_ atmosphere (100 mL min^−1^) from room temperature to 800 °C (10 °C min^−1^ heating rate).

### 2.4. Mechanical properties

Mechanical properties of the bionanocomposite films were determined by tensile test using a Model 3345 Instron Universal Testing Machine (Instron Engineering Corporation Canton). A minimum of 3 test pieces of 45 × 10 mm were evaluated for each sample. Thickness of each piece was measured using a thickness gauge Mitutoyo No. 2118-50 Dial Indicator 0.001–5 mm. Initial grips separation and crosshead speed were fixed to 20 mm and 15 mm min^−1^, respectively. Young modulus (GPa), tensile strength (MPa) and elongation at break (%) were calculated from stress–strain curves. Tukey's multiple comparison test (*α* = 0.05) was used to determine differences between the mean values of the mechanical properties of the samples.

### 2.5. Water vapor sorption isotherms

Water vapor sorption isotherms were determined between 0% and 95% relative humidity (RH) at 25 °C using a dynamic water vapor sorption equipment (Aquadyne DVS, Quantachrome Instruments). The GAB model was used to fit the recorded experimental adsorption isotherms. This model considered that the total water uptake (*C*_GAB_) is a function of water activity (*a*_w_ = RH/100) according to eqn (1):^20^1
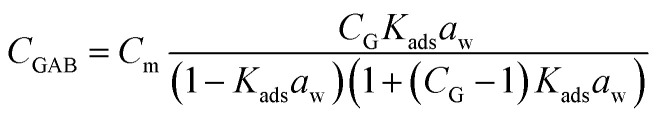
where: *C*_m_ is the amount of water adsorbed onto the monolayer (monolayer capacity); *C*_G_ is related to the strength of bound water to the primary binding sites (Guggenheim constant), and *K*_ads_ refers to the adsorption enthalpy difference between the first layer and the following.

### 2.6. Gas barrier properties

The barrier properties of the bionanocomposite films were evaluated by determining their water vapor and oxygen permeability. The water vapor permeability (WVP) was determined gravimetrically based on ASTM E96 standard (procedure for desiccant method). The bionanocomposite films were mounted on the perforated lid of test cells using aluminum foil masks, with an inner diameter of 1 cm. Dry silica-gel was used to fill the test cells before being sealed. Then, test cells were placed into a humidity chamber at 75% relative humidity and room temperature and were weighed each 24 hours during at least 4 days. Experiments were carried out by triplicate for each bionanocomposite film. The slopes of the weight gain *versus* time curves were divided by the exposed film area to calculate the water vapor transmission rate (WVTR). Then, WVP was determined taking into account the thickness of each film (*L*, in mm) and the partial water vapor pressure difference across both sides of the film (Δ*P*, in kPa), according to eqn (2):2
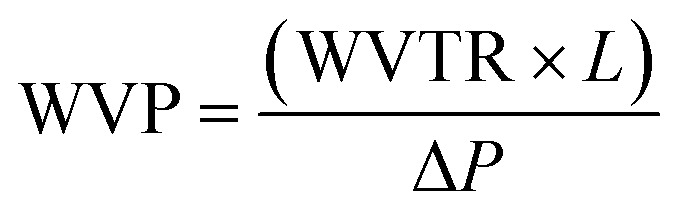


Oxygen permeability measurements were carried out at the ICTP (Institute of Polymer Science and Technology, CSIC), based on the constant volume pressure method. In this case, only one measurement was carried out for each of the selected samples. The bionanocomposite films were placed in the test cell dividing the cell in two zones called high and low pressure chambers. The device was connected to a high-vacuum system (turbomolecular pump), temperature sensors and pressure gauge, and it was placed in a thermostatic bath at 30 °C. The pressure in the high pressure chamber was measured using a pressure sensor (range of 0–1 bar absolute) from Gometrics and maintained at 1 bar, and the amount of gas (oxygen) passing through the bionanocomposite film was monitored as a function of time using a MKS-6 pressure transducer with an interval of 0–0.0133 bar. The value of the oxygen permeability was calculated as follows (eqn (3)):3
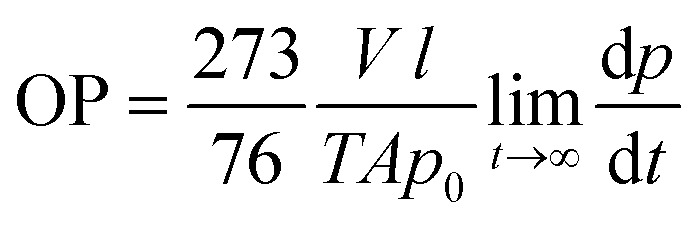
where: *p*_0_ and *p* are the pressure in the high and low pressure chambers, respectively (in cm Hg); *T* is the temperature of the experiments (in K); *V* is the volume of the low pressure chamber (in cm^3^), *A* is the permeation area of the film (in cm^2^) and *l* is the film thickness (in cm). Thus, OP is expressed in barrers (1 barrer = 10^−10^ cm^3^ (STP) cm cm^−2^ s^−1^ cm Hg^−1^), and then converted to mL m m^−2^ d^−1^ Pa^−1^.

The diffusion coefficient (*D*, in cm^2^ s^−1^) was determined from the film thickness (*l*) and the delay time (*θ*) according to eqn (4).4
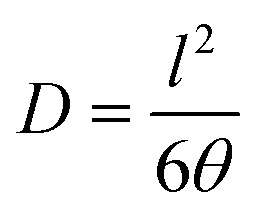


Finally, the apparent solubility coefficient (*S*, in cm^3^ (STP) cm^−3^ cm Hg) was obtained dividing OP (in barrers) by diffusion coefficient (in cm^2^ s^−1^), according to eqn (5):5
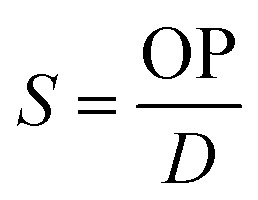


### 2.7. Water contact angle

Water contact angle measurements were carried out using the sessile drop method in a DataPhysics Instrument OCA 15 plus running on SCA 20/21 software. Droplets of deionized water (5 μL) were mechanically dropped onto the bionanocomposite film surface and the corresponding contact angles were determined using the Young–Laplace method. The average values were obtained at five different positions for each sample.

### 2.8. Light barrier properties and color evaluation

To determine the light barrier properties of the bionanocomposite films, their optical transmittance was measured in the wavelength range between 200 and 900 nm, using a UV-1201 spectrophotometer (Shimadzu) equipped with an integrating sphere.

Color changes were also evaluated based on CIE L*a*b* coordinates, using an ELREPHO 070 spectrophotometer (Lorentze and Wettre). Film samples were placed on top of a white paper with the following CIE L*a*b* coordinates: L* = 97.89, a* = −0.26 and b* = 2.43. The total color changes were determined according to eqn (6):6
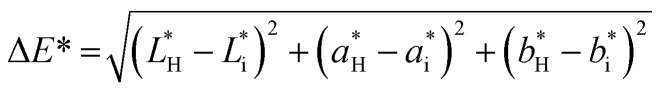
where 
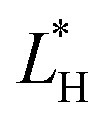
, 
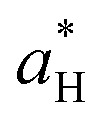
 and 
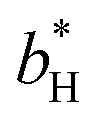
 are the color coordinates of HPMC sample (H) and 
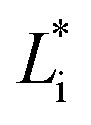
, 
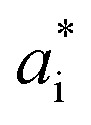
 and 
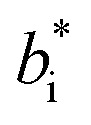
 are the color coordinates of each bionanocomposite film sample.

### 2.9. Antioxidant capacity

The antioxidant capacity of the bionanocomposite films was determined following the ABTS^+^˙ method described by Re *et al.*^21^ The ABTS^+^˙ solution was produced by mixing a 7 mM ABTS aqueous solution with potassium persulfate (2.45 mM final concentration) and allowing them to react for 16–24 hours in the dark and at room temperature. Afterwards, the absorbance of the ABTS^+^˙ solution was adjusted to 0.70 ± 0.02 at 734 nm by diluting with phosphate buffer (5 mM, pH 7.4). Then, 1 mL of the resulting ABTS^+^˙ solution was mixed with 10 μL of film solution (prepared by dissolving 15 mg of each bionanocomposite film in 500 μL of ultrapure water) and thoroughly mixed in a vortex (10 s). Right after, the changes in absorbance of the reaction mixture were measured during 6 min at 734 nm, using a UV-1201 spectrophotometer (Shimadzu). The percentage of inhibition (% I) of each sample was calculated according to eqn (7), performing at last three measurements for each film solution.7
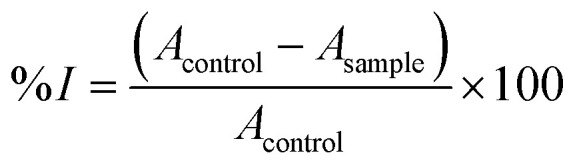
where *A*_control_ is the absorbance with the blank (water), and *A*_sample_ is the absorbance with the sample after 6 min.

Trolox (6-hydroxy-2,5,7,8-tetramethylchroman-2-carboxylic acid) was used as standard, following the same procedure to determine the % of inhibition for Trolox solution (dissolved in phosphate buffer) with concentration between 0.25 and 1.50 mM. Thus, the antioxidant capacity of the bionanocomposite films was expressed as mg of Trolox equivalent (TE) per g lignin.

### 2.10. Antibacterial activity

The antibacterial activity of the bionanocomposite films was evaluated against *Escherichia coli* (CECT 433) and *Staphylococcus aureus* (CECT 231). Inoculum solutions were prepared by overnight cultivation of one bacterial colony forming in Lennox Luria Bertani broth (LB) and nutrient broth (NB) for *E. coli* and *S. aureus* respectively, at 37 °C and 140 rpm. Each bionanocomposite film (20 mg) was placed in a sterile tube with 2 mL of the corresponding sterilized culture medium (LB for *E. coli* and NB for *S. aureus*). Controls with 2 mL culture medium but without film were also prepared. 40 μl of the corresponding overnight inoculum were added to each tube and they were incubated for 24 h at 37 °C and 140 rpm. After the incubation time, serial dilutions of the cultures were prepared in sterilized phosphate buffer (PBS) from 10^−1^ to 10^−8^ and 100 μl of each of them were evenly spread on agar medium plates under sterile conditions. The inhibition effect was evaluated by counting the number of bacterial colonies after incubation at 37 °C during 24 hours and it was expressed as the percentage of reduction in the number of colonies with respect to the control experiment with HPMC film. At least three experiments were performed for each sample.

## 3. Results and discussion

### 3.1. Morphology of the HPMC/montmorillonite/lignin bionanocomposite films

**Fig. 2:**
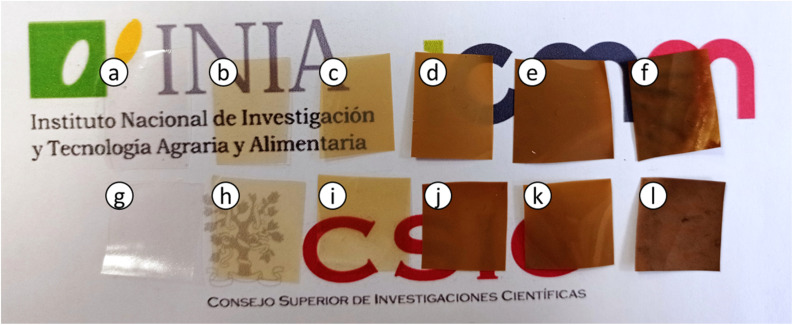
Appearance of the bionanocomposite films: (a) HPMC, (b) H-0.5L, (c) H-1L, (d) H-3L, (e) H-5L, (f) H-10L, (g) H-3M, (h) H-3M-0.5L, (i) H-3M-1L, (j) H-3M-3L, (k) H-3M-5L, (l) H-3M-10L.

HPMC films were prepared with different lignin content from 0 to 10% as indicated in Table 1, obtaining homogeneous and uniform films, except for 10% lignin content, in which phase separation was observed (Fig. 2 and Fig. SI.1a–f in ESI†). H-10L films also presented higher thickness (90 ± 7 μm *vs.* 55 ± 4 μm) and lower apparent bulk density (0.89 ± 0.02 g cm^−3^*vs.* 1.14 ± 0.11 g cm^−3^) than films with lower lignin content (0–5%), which indicates higher porosity probably related to less compact structures with lower hydrogen bonding^22^ due to too high lignin content. Related to color, lignin gave brown color to the bionanocomposite films, which increased as expected when lignin content increased (Fig. 2). Despite this, films remained transparent for lignin content up to 1%, as will be discussed later in Section 3.7. When films were reinforced with 3% of montmorillonite, no significant changes in film appearance were found (Fig. 2 and SI.1a–f in ESI†). Similarly to sample H-10L, H-3M-10L films presented higher thickness (92 ± 9 μm *vs.* 55 ± 4 μm) and lower apparent bulk density (0.86 ± 0.08 g cm^−3^*vs.* 1.15 ± 0.10 g cm^−3^) than H-3M films with 0–5% lignin content, being phase separation clearly noticeable.

**Fig. 3:**
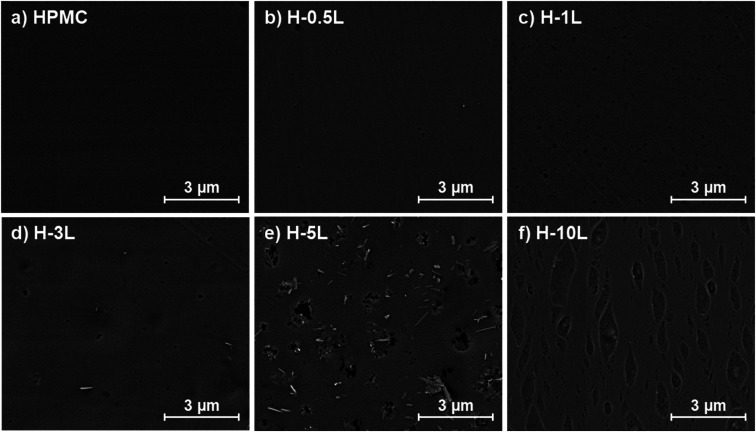
Study of the film morphology by FE-SEM: surface images of the bionanocomposite films without montmorillonite, with different lignin content: 0% (a), 0.5% (b), 1% (c), 3% (d), 5% (e) and 10% (f).

**Fig. 4:**
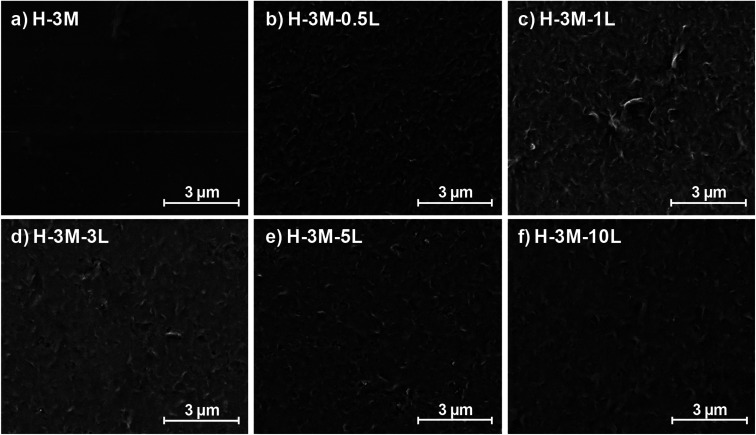
Study of the film morphology by FE-SEM: surface images of HPMC films with 3% montmorillonite, with different lignin content: 0% (a), 0.5% (b), 1% (c), 3% (d), 5% (e) and 10% (f).

FE-SEM images showed the surface of the different films without montmorillonite (Fig. 3) and with montmorillonite (Fig. 4). HPMC film presented a homogeneous and flat surface that remained almost similar when 0.5% lignin was added (H-0.5L). However, some lignin dots were observed in H-0.5L surface, which increased in number and size when the lignin content increased. Up to 3 and 5% lignin content, some individual and staked rods were also found. EDX analysis (Fig. SI.2 in ESI†) revealed that these rods correspond to lignin, in the presence of sodium, since the O/C ratio was 0.26, similar to that previously reported for other lignins,^23,24^ and lower than that corresponding to HPMC (0.54). However, these lignin rods were not observed in H-10L surface, where elongated marks were found instead, probably due to phase separation, observed also without microscopy.

**Fig. 5:**
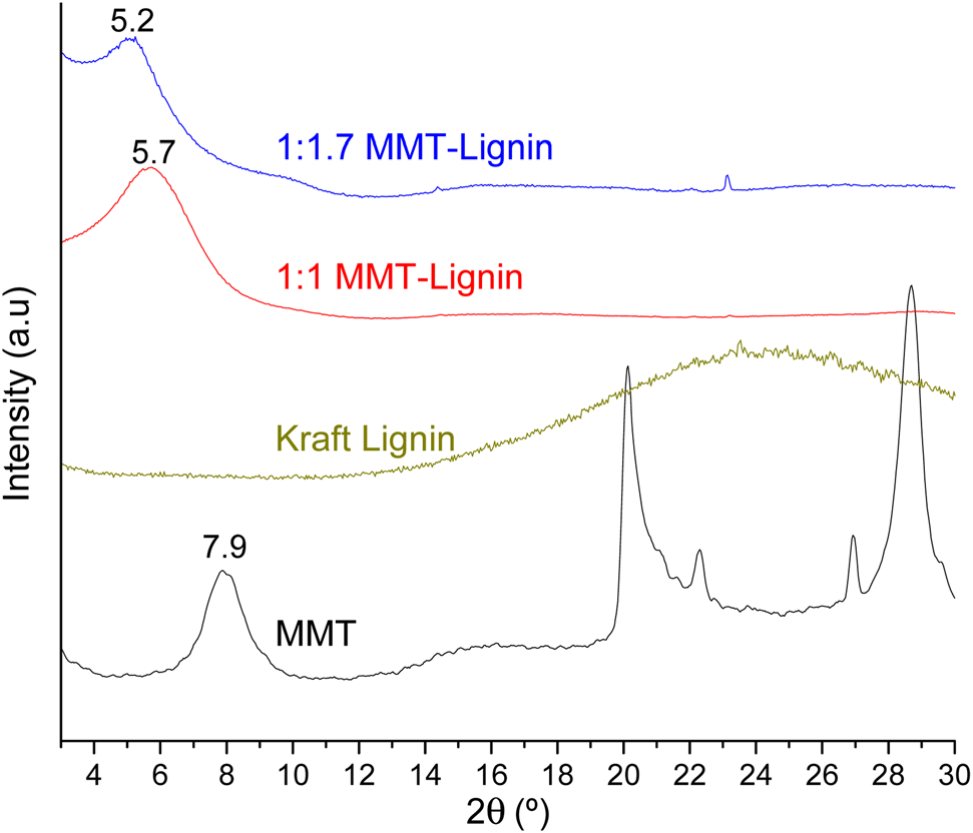
XRD patterns of montmorillonite (MMT), lignin, 1 : 1 MMT–lignin hybrid and 1 : 1.7 MMT–lignin hybrid.

**Fig. 6:**
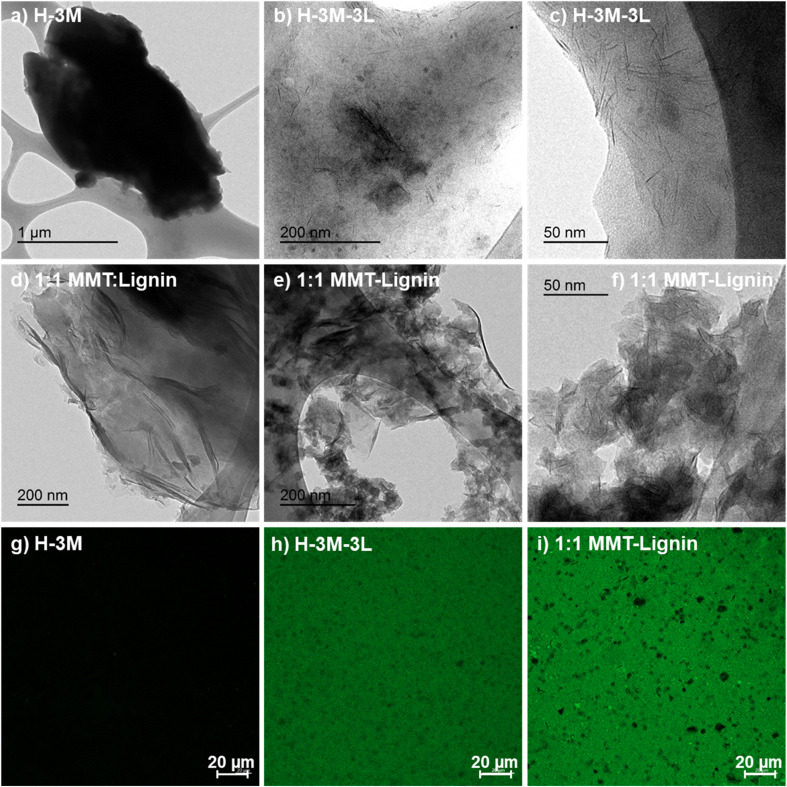
Study of the film morphology by TEM: images of HPMC films with 3% montmorillonite without lignin (a), HPMC with 3% montmorillonite and 3% lignin (b and c), and 1 : 1 MMT–lignin hybrid (d–f); and confocal images of HPMC films with 3% montmorillonite without lignin (g), HPMC with 3% montmorillonite and 3% lignin (h), and 1 : 1 MMT–lignin hybrid (i).

**Fig. 7:**
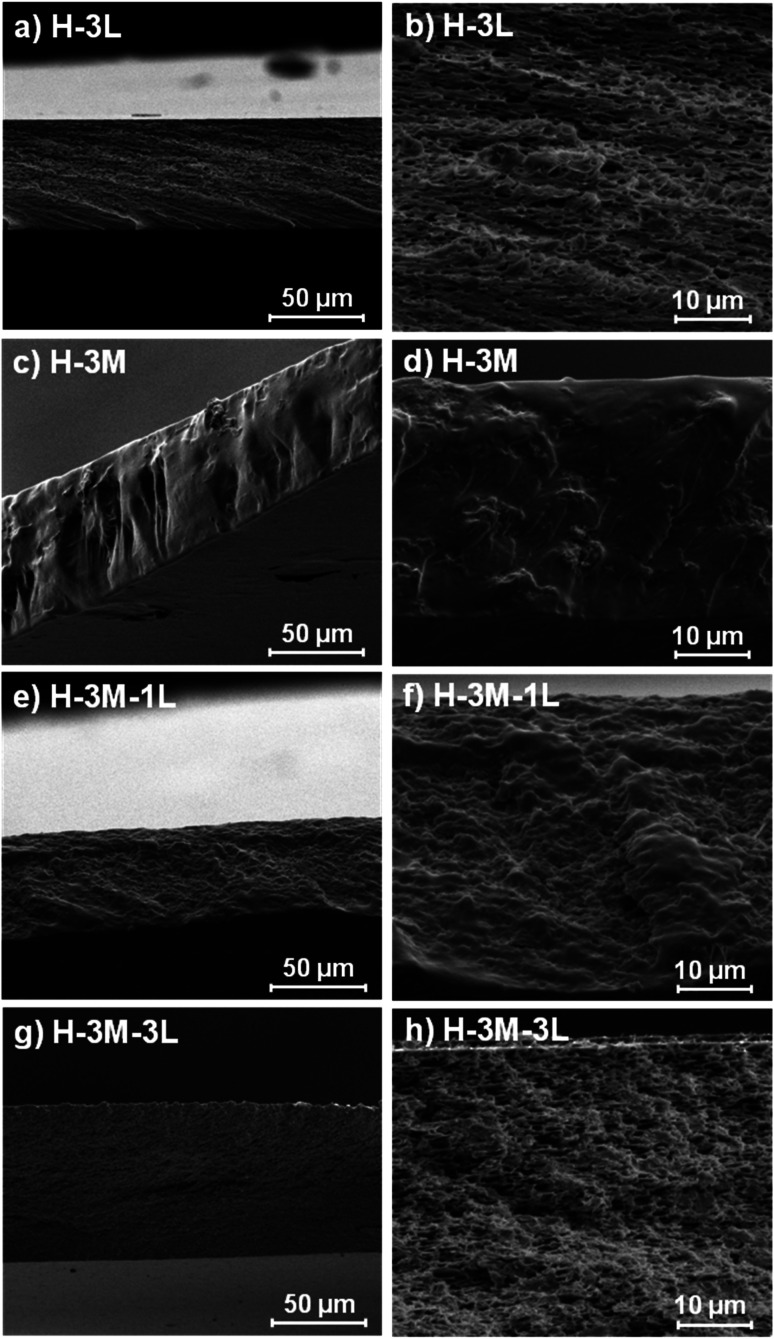
FE-SEM images of the cross-section of H-3L (a and b), H-3M (c and d), H-3M-1L (e and f) and H-3M-3L (g and h) bionanocomposite films.

When montmorillonite was incorporated to the bionanocomposite films, rougher surfaces were detected, in agreement with Darder *et al.*^16^ who observed several spots embedded in the polymer when gentamicin–montmorillonite hybrid was integrated into HPMC films. Interestingly, lignin rods were not observed until lignin content increased up to 10%. Furthermore, FE-SEM images of H-3M-10L (Fig. 4f) did not show a clear phase separation, contrarily to H-10L (Fig. 3f), but TEM images did confirm the presence of areas with lignin aggregates (Fig. SI.3a†), areas with high montmorillonite concentration (Fig. SI.3b†) and areas showing the presence of both compounds (Fig. SI.3c†). These results are in agreement with the phase separation observed in Fig. 2 and SI.1.† The better integration of lignin into the HPMC when montmorillonite was present, could be attributed to the role of the layered clay preventing or hindering lignin aggregates, likely due to lignin intercalation into montmorillonite. XRD diffractograms (Fig. 5) show a shift of the (001) reflection of pristine montmorillonite from 7.9° to 5.7° in 1 : 1 MMT–lignin hybrid (incorporated to H-3M-3L) and to 5.2° in 1 : 1.7 MMT–lignin hybrid (incorporated to H-3M-5L), pointing to the intercalation of lignin in the phyllosilicate. These 2*θ* values indicated an increase in the basal spacing (*d*-spacing) from 1.12 nm to 1.55–1.69 nm, which correspond to interlayer distances of 0.59–0.73 nm, which are compatible with the polymer thickness. Other authors have also reported the expansion of interlayer spacing of montmorillonite caused by lignin or other macromolecular organic compounds with amphiphilic property, which reduce the interaction force of free hydrated ions in montmorillonite sheets and increase the *d*-spacing by the action of ion dipoles.^25,26^ Furthermore, Yue *et al.*^27^ reported that this expansion could be extended until a complete dispersion of exfoliated montmorillonite in the polymer matrix in the presence of alkali lignin in alkaline conditions. The exfoliation of montmorillonite could be the reason of the disappearance of most of the XRD diffraction peaks ascribed to montmorillonite in MMT–lignin hybrids (Fig. 5), observing only the (001) reflection shifted to lower 2theta angle which is indicative of intercalation. Thus, the absence of other rational reflections and the enlargement of the (001) peak are indicative of partial delamination and disorganization in the stacking. TEM analysis confirmed exfoliation: the typical montmorillonite dark particles composed of several layers found in H-3M film (Fig. 6a) were not found in MMT–lignin hybrid (Fig. 6d–f) where some smaller and lighter particles (corresponding to only a few layers packed together or even individual layers) were found along with individual layers rolled up on themselves. Similar changes were observed by Letaïef *et al.*^28^ related with delamination of silicate layers. When the MMT–lignin hybrid was incorporated to HPMC matrix (H-3M-3L, Fig. 6b and c) the extent of montmorillonite exfoliation increased observing mostly individual rolled layers along with few individual unrolled layers (smaller and lighter than those observed in MMT–lignin hybrid). The absence of the XRD diffraction peaks ascribed to montmorillonite in the HPMC/montmorillonite/lignin bionanocomposite (Fig. SI.4 in ESI†) could be related to this exfoliation. However, neither the XRD pattern of H-3M sample showed peaks ascribed to montmorillonite whereas particles of packed montmorillonite were clearly observed by TEM (Fig. 6a). Therefore, the absence of montmorillonite signals in XRD patterns is most likely due to a dilution effect since the bionanocomposite contained only a 3% of clay. In conclusion, the presence of lignin assisted by ultrasound treatment caused partial intercalation and montmorillonite exfoliation in MMT–lignin hybrids, resulting in almost complete exfoliation when they were incorporated into the HPMC matrix, achieving a very good distribution of both lignin and montmorillonite into the matrix. Thus, confocal microscopy showed a very homogeneous distribution of both lignin (with green fluorescence) and montmorillonite (dark spots) in the HPMC matrix (H-3M-3L sample, Fig. 6h), while larger and slightly less homogeneously distributed montmorillonite spots were observed in MMT–lignin hybrid (Fig. 6i). Therefore, it could be concluded that in the present case, lignin can act like a tensioactive additive improving the compatibility and distribution of the clay into the polymer matrix, which could be of great importance in the future developments of bio-based-organoclays. The surfactant role of lignin was also confirmed by analyzing the FE-SEM images of the cross-section of the films. Thus, when lignin was present in the bionanocomposite film, regardless of the presence of montmorillonite (Fig. 7a and b for H-3L and 7g and h for H-3M-3L), the aspect is very homogeneous but with a texture like a foam in comparison to the more compact aspect of samples without lignin (Fig. 7c and d for H-3M). When the amount of lignin was reduced to 1%, a less foamy cross-section, with less air bubbles or gaps was observed (Fig. 7e and f for H-3M-1L), highlighting the surfactant effect of lignin.

### 3.2. FTIR characterization

**Fig. 8:**
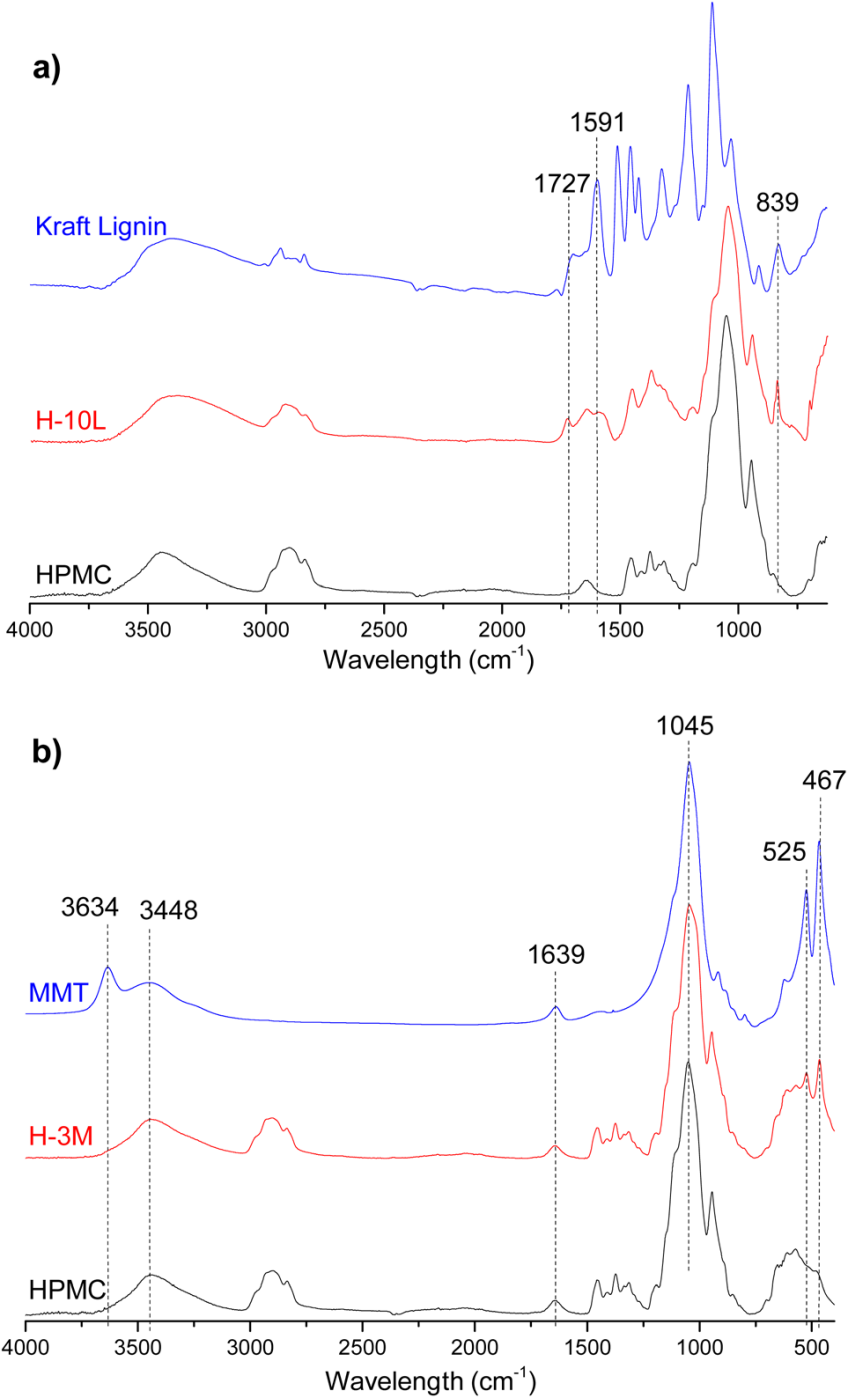
Fourier transform infrared (FTIR) spectra of (a) kraft lignin and HPMC films without lignin (HPMC) and with 10% lignin (H-10L) and (b) montmorillonite (MMT) and HPMC films without MMT (HPMC) and with 3% MMT (H-3M).

FTIR spectrum of HPMC film (Fig. 8) showed typical vibration bands for this polymer: 3452 cm^−1^ assigned to stretching of the hydroxyl groups (*ν*_O–H_), 2901 and 2836 cm^−1^ corresponding to stretching vibration related to *ν*_C–H_ bonds, 1637 cm^−1^ assigned to *δ*_H–O–H_ bending of water, 1456 and 1374 cm^−1^ corresponding to –CH_2_ and –OH groups respectively, and 1052 and 944 cm^−1^ assigned to *ν*_C–O_ stretching vibrations of C–OH groups.^29,30^Fig. 8a shows a comparison between FTIR spectra of HPMC, kraft lignin and a HPMC film with the higher lignin content (H-10L). Due to overlapping with HPMC signals, only one of the three bands corresponding to vibrations of aromatic skeleton of lignin were appreciated in H-10L spectrum: 1591 cm^−1^ compared to 1591, 1511 and 1457 cm^−1^ in kraft lignin spectrum.^31^ Similarly, only two of the other typical bands of kraft lignin were observed in H-10L: 1727 cm^−1^ assigned to unconjugated *ν*_C=O_ groups stretching from lignin oxidation and 839 cm^−1^ assigned to *δ*_C–H_ out of plane bending in positions 2 and 6 of aromatic ring,^31^ suggesting a high S lignin content typical of hardwoods species such as *E. globulus*. The intensity of these bands decreased when decreasing lignin content, not being detected when lignin content was lower than 3% (Fig. SI.5 in ESI†) due to dilution effect in the HPMC matrix. Similarly, most of the bands corresponding to pristine montmorillonite (3634 and 3448 cm^−1^ associated with stretching vibration modes of Al, Mg(OH) and –OH groups of interlayer water, 1639 cm^−1^ assigned to –OH bending mode of water and 1045 cm^−1^ corresponding to *ν*_Si–O_ stretching and *δ*_Si–O–Si_ bending vibrations)^9,16^ were not detected in the FTIR spectra of bionanocomposite films with 3% montmorillonite (Fig. 8b and SI.6 in ESI†), which could be attributed to signal overlapping and dilution effects. Only bands at 525 and 467 cm^−1^ corresponding to *ν*_Al–O_ and *ν*_Mg–O_ stretching vibrations, respectively,^9^ were observed in the bionanocomposites containing montmorillonite. Due to signal overlapping, it is very difficult to detect possible interactions between the three components integrated in the bionanocomposite films.

### 3.3. Thermal stability

Thermal stability of the bionanocomposite films was studied by TGA. The corresponding curves can be found in ESI (Fig. SI.7a†) and the characteristic parameters inferred from these curves are summarized in Table 2. A first weight loss of 3–5% was observed between 52 and 61 °C in all samples due to moisture, similar to that observed by Mondal *et al.*^10^ in HPMC films with different montmorillonite content (0–7%). Pure HPMC sample presented its major weight loss at 361 °C, corresponding to structural decomposition of HPMC.^10^ This degradation temperature (*T*_deg_) shifted to lower values when lignin was added, observing lower *T*_deg_ and *T*_on_ values by increasing lignin content. Although kraft lignin presented its major weight loss at a temperature close to that of HPMC (*T*_deg_ of lignin 337 °C), its degradation took place in a wider range of temperature, starting at lower temperature than HPMC (*T*_on_ of 204 °C compared to 339 °C for HPMC) and ending at higher temperature (*T*_off_ of 490 °C *vs.* 378 °C) (Fig. SI.7b in ESI†). Thus, when lignin was incorporated in the HPMC matrix, a reduction in thermal stability was found. Similar results were found by Shankar *et al.*,^32^ Xiong *et al.*^33^ and Tedeschi *et al.*^34^ when incorporating lignin into agar, poly(vinyl alcohol) or xylan–cellulose matrices, respectively. Interestingly, when lignin content was higher than 3%, a small weight loss of approximately 2–6% was observed around 135 °C, probably due to the evaporation of remaining water associated with lignin and to the dehydration of hydroxyl groups from benzyl groups (previously reported at temperature between 130 and 150 °C).^33,35^ The incorporation of lignin in amounts higher than 1% increased the char residue found at 800 °C from 12 to 14–21% due to the high char residue of kraft lignin at this temperature (38%).

**Table 2:** Characteristic parameters from thermogravimetric analysis (TGA) of the bionanocomposite films: degradation temperatures (*T*_deg_), temperatures at which degradation of the main peaks begins (*T*_on_) and ends (*T*_off_), loss of weight corresponding to each peak (Δ*W*) and char residue at 800 °C (CR)

Sample	*T*_deg_ (°C)	*T*_on_ (°C)	*T*_off_ (°C)	Δ*W* (%)	CR (%)
HPMC	54/361	339	378	3/85	12
H-0.5L	55/358	327	377	4/84	12
H-1L	60/357	315	375	4/82	14
H-3L	56/135/326	277	368	4/2/79	15
H-5L	61/135/307	279	354	3/2/74	21
H-10L	52/135/324	289	358	5/6/69	20
H-3M	61/359	340	375	4/84	12
H-3M-0.5L	56/358	324	377	4/81	15
H-3M-1L	61/356	316	373	4/80	16
H-3M-3L	60/135/325	279	366	4/1/74	22
H-3M-5L	58/132/310	281	355	5/2/70	24
H-3M-10L	52/131/312	284	338	5/5/64	25

When 3% of montmorillonite was added, no significant changes in *T*_deg_, *T*_on_ and *T*_off_ were found compared to corresponding films without clay. Contrarily, Mondal *et al.*^10^ reported a slight increase from 334 to 337 °C when adding 3% of montmorillonite to HPMC films, which increased up to 355 °C for 7% of montmorillonite. Nevertheless, these *T*_deg_ were lower than those reported here for HPMC and H-3M (359–361 °C). These different results could be due to differences in the experimental preparation of the films or in the raw materials used. Finally, an increase of approximately 2–5% in the char residue at 800 °C was found in most of the films with montmorillonite due to the incorporation of this inorganic compound.

### 3.4. Mechanical properties

**Fig. 9:**
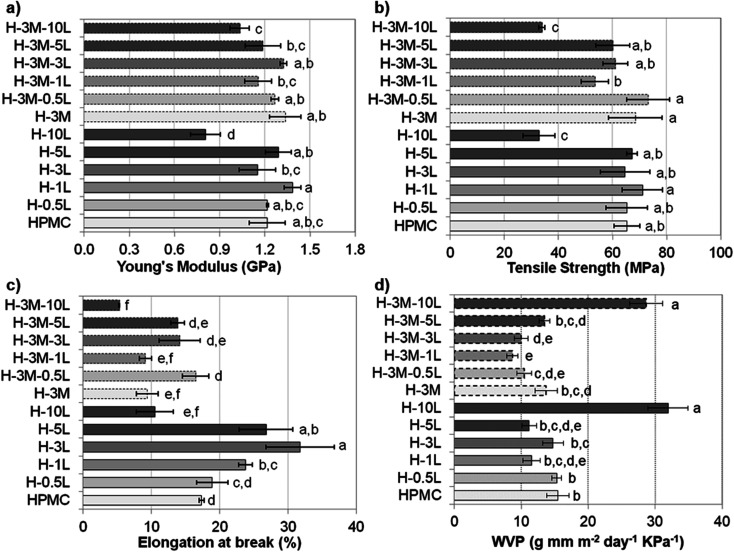
Mechanical and barrier properties of bionanocomposite films: (a) Young's modulus, (b) tensile strength, (c) elongation at break and (d) water vapor permeability (WVP). According to Tukey's multiple comparison test, values followed by the same letter in the same graphic do not differ significantly (*p* < 0.05).

Mechanical properties of the bionanocomposite films were evaluated by tensile test (stress–strain curve are shown in Fig. SI.8, in ESI†), determining Young's modulus, tensile strength and elongation at break (Fig. 9). HPMC films showed a Young's modulus of 1.2 ± 0.1 GPa, a tensile strength of 65 ± 5 MPa and an elongation at break of 17.4 ± 0.4%, all in the range previously reported for HPMC films (0.60–2.5 GPa, 21–75 MPa and 6.6–31%, respectively).^6–10,16^ In fact, all the bionanocomposite films studied in this work (with lignin and/or montmorillonite), presented values of mechanical properties in these ranges, indicating their suitability for food packaging applications.^36^

According to Tukey's multiple comparison test (*p* < 0.05), the addition of lignin and/or montmorillonite did not cause significant changes in the values of Young's modulus and tensile strength, except for a lignin content of 10%. Thus, both H-10L and H-3M-10L showed a significant drop in mechanical properties, likely due to phase separation and a less compact structure with lower hydrogen bonding according to their higher thickness (90–92 μm) compared to the rest of the films (55 ± 4 μm), as it was indicated above. Nevertheless, when montmorillonite was not present, a certain trend could be observed, which indicated an improvement in Young's modulus and tensile strength when lignin content increased up to 1%, followed by a slight reduction for 3% and 5% lignin. A similar trend was observed when lignin was added to other polymeric matrixes such as poly(vinyl alcohol)^33^ or agar.^32^ The increase in mechanical properties could be related to a good dispersion of lignin in the polymer matrix.^32,37^ Thus, homogeneous surfaces were observed in the FE-SEM images of HPMC films with lignin content up to 1%, while lignin aggregates were found for higher lignin contents (Fig. 3). On the other hand, a slight but not statistically significant increase in both Young's modulus and tensile strength was observed when 3% montmorillonite was incorporated. In this regard, Moura *et al.*^9^ and Mondal *et al.*^10^ reported clear improvements in both parameters when 1–7% montmorillonite was added to HPMC films.

Contrarily, elongation at break showed a clearer effect of lignin and montmorillonite addition. Thus, when montmorillonite was not present in the bionanocomposite, a clear increase was observed by lignin addition up to 3%, indicating that lignin might act as a plasticizer agent. A similar effect has been observed when other antioxidant agents such as essential oils were added to HPMC films.^7,8^ In the same way, lignin has been reported to act as a plasticizer agent in blend films with fish gelatin, alginate, starch or soy protein, but in some cases only when added in moderate concentration.^38,39^ Thus, for further increases in lignin content up to 10%, a significant reduction in their plastic behavior was observed. On the other hand, the addition of montmorillonite (3%) caused a reduction in elongation at break, which partially hinders the effect of lignin addition. This reduction in elasticity could be explained by the more rigid structure of the layered clay, reducing the mobility of the biopolymer chains after their assembling to the clay. Nevertheless, controversial results have been reported to this regard. Thus, Moura *et al.*^9^ and Darder *et al.*^16^ also reported a reduction in elongation and an increase in both tensile strength and Young's modulus when incorporating montmorillonite (2,5–4.0%) or gentamicin–montmorillonite hybrid (2.4–12.9% clay content) in HPMC, respectively. However Mondal *et al.*^10^ reported a significant increase in the three parameters when a 3% of clay was added.

### 3.5. Water vapor sorption isotherms

**Fig. 10:**
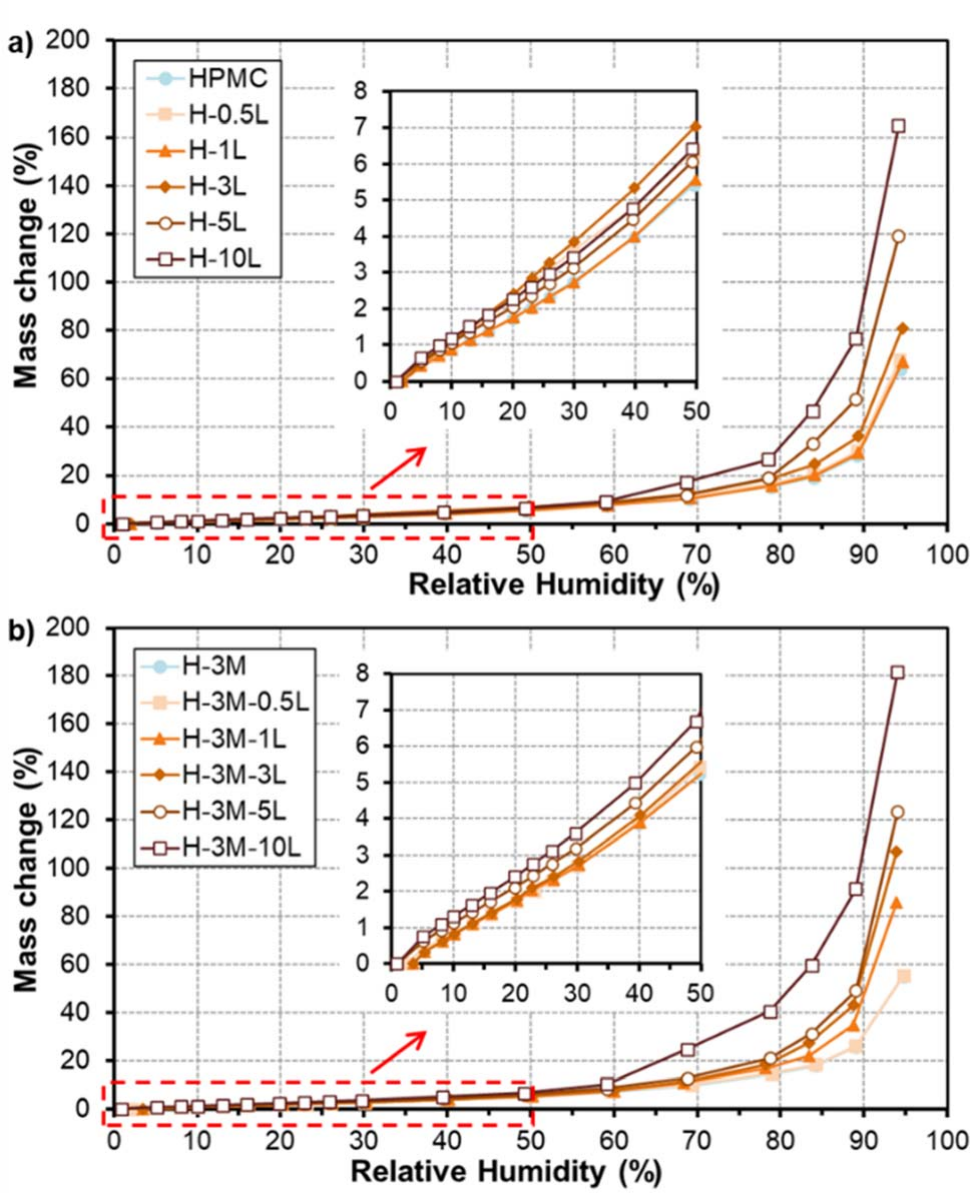
Water sorption isotherms of HPMC films without montmorillonite (a) and with montmorillonite (b) and different lignin content.

Fig. 10 shows the water vapor sorption isotherms of the bionanocomposite films. All the films have a similar behavior in which moisture content of the film slowly increased for relative humidity values lower than 60%. However, large mass gains were found for higher humidity values, due to film swelling and penetration of water into the HPMC network, partially dissolving the film to transform it into a gel.^40,41^ The presence of a plasticizer can enhance the film moisture content, due to additional space between the polymer chains.^8^ This could be the reason for the higher water vapor sorption observed for films with high lignin content, since lignin could be acting as plasticizer as mentioned above. Regarding the effect of montmorillonite addition, no big changes were observed in HPMC isotherms, in agreement with results reported by Darder *et al.*^16^ after the incorporation of gentamicin–montmorillonite hybrid. Thus, a reduction in moisture absorption of only 5% was found comparing H-3M with pure HPMC films at RH of 70%, in contrast to the higher reduction observed by Mondal *et al.*^10^ for similar films at 73% RH (reduction of 22.77% in HPMC films with 3% montmorillonite compared to pure HPMC film).

In order to better compare the water vapor sorption properties of the films, isotherms curves were fitted to GAB model (Table 3). This model considers that water molecules adsorb layer by layer on adsorption surface (external surfaces of specific sites or internal surfaces of micro-pores/cavities). The first layer of water covers the surface unevenly and is tightly bound in a monolayer, while subsequent layers display increasingly bulk-like properties. The monolayer capacity (*C*_m_) represents the amount of water adsorbed onto one layer, the Guggenheim constant (*C*_G_) measures the strength of bound water to the primary binding sites and *K*_ads_ refers to the adsorption enthalpy difference between the first layer and the following. The monolayer capacity of HPMC film (3.0 g/100 g dry solid) was very similar to that reported previously for HPMC film (2.9 g/100 g dry solid) by Villalobos *et al.*^41^ According to the estimated parameters (Table 3), adsorption enthalpy difference did not change significantly with the addition of lignin nor montmorillonite. However, an increase in the monolayer capacity (*C*_m_) and a reduction in the strength of bound water (*C*_G_) were observed for increasing lignin content, especially for 5–10% lignin. On the other hand, the addition of montmorillonite did not show a clear effect on the monolayer capacity but it reduced the strength of bound water to the primary binding sites. Similarly, Mondal *et al.*^10^ reported that in HPMC/montmorillonite films the free water molecules did not interact as strongly as in HPMC film due to the formation of hydrogen bonds between HPMC and montmorillonite.

**Table 3:** Sorption parameters of GAB models, determined from water vapor sorption isotherms for the bionanocomposite films

Sample	*C*_m_	*C*_G_	*K*_ads_	*R* ^2^
HPMC	3.00 ± 0.10	4.88 ± 1.49	1.011 ± 0.001	0.9977
H-0.5L	3.25 ± 0.12	6.68 ± 2.44	1.010 ± 0.001	0.9969
H-1L	3.18 ± 0.10	4.19 ± 1.00	1.007 ± 0.001	0.9984
H-3L	3.89 ± 0.08	4.72 ± 0.84	1.007 ± 0.001	0.9992
H-5L	5.25 ± 0.22	1.24 ± 0.26	1.018 ± 0.001	0.9990
H-10L	10.25 ± 0.90	0.44 ± 0.09	1.004 ± 0.003	0.9991
H-3M	3.10 ± 0.08	4.02 ± 0.73	0.997 ± 0.001	0.9989
H-3M-0.5L	3.17 ± 0.09	3.99 ± 0.81	0.996 ± 0.002	0.9986
H-3M-1L	3.40 ± 0.07	3.06 ± 0.47	1.023 ± 0.001	0.9995
H-3M-3L	4.20 ± 0.06	1.85 ± 0.17	1.024 ± 0.001	0.9998
H-3M-5L	4.57 ± 0.07	2.12 ± 0.24	1.025 ± 0.001	0.9998
H-3M-10L	13.54 ± 1.2	0.50 ± 0.10	0.994 ± 0.004	0.9990

### 3.6. Gas barrier properties

As indicated in the Introduction, HPMC, like other polysaccharides, is water sensitive showing poor water vapor barrier properties and causing high oxygen permeability when relative humidity increases.^8^ Taking into account the importance of barrier properties in food packaging, several approaches have been published to address this constraint, such as the incorporation of hydrophobic surfactants, lipid compounds or clays.^6–12^ Thus, an 11.4% reduction in water vapor permeability (WVP) was achieved when 3% of montmorillonite was added (H-3M compared to HPMC film, Fig. 9d). This improvement in moisture barrier can be ascribed not only to an increase in water contact angle (45.9 ± 2.9° for H compared to 54.6 ± 3.1° for H-3M) but also to an increased tortuosity in the water vapor diffusion path caused by the presence of impermeable clay layers in the polymer matrix. Similar results were found by Mondal *et al.*,^10^ reporting a reduction of 12.5%. Nevertheless, higher reductions (up to 63%) were reported by Moura *et al.*^9^ for HPMC with 2.5% montmorillonite. This higher reduction could be due to higher initial WVP for HPMC control film (19.1 g mm kPa^−1^ d^−1^ m^−2^ compared to 15.5 g mm kPa^−1^ d^−1^ m^−2^ in our work).

On the other hand, when lignin was incorporated into the bionanocomposite an improvement in moisture barrier was also found for most of the samples (Fig. 9d). This could be related to the hydrophobicity of lignin, as also revealed by the increase in water contact angle observed in the films when lignin was incorporated (45.9 ± 2.9° for H compared to 63.3 ± 2.5° for H-3L). Shankar *et al.*,^32,42^ Chen *et al.*^43^ and Tedeschi *et al.*^34^ also reported reductions in WVP when incorporating lignin to agar, polylactic acid (PLA), chitosan or xylan–cellulose matrixes, respectively. These authors correlated the improvements in moisture barrier to strong intermolecular interaction between the biopolymer matrix and the lignin. However, when lignin content increases up to certain content, an increase in WVP has been reported probably due to lignin aggregation.^32,44^ This would explain the lack of improvement in moisture barrier for lignin content higher than 1%, in agreement with lignin rods and dots observed in FE-SEM images for H-3L and H-5L (Fig. 3). The large increase in WVP found for H-10L could be due to the observed phase separation and the higher porosity of this film, indicated above, which lead to less compact structures.

When both lignin and montmorillonite were present in the bionanocomposite films, a similar trend due to lignin addition was observed (Fig. 9d). Furthermore, a synergistic effect took place, showing a reduction in WVP of 25–32% in most of the cases, comparing a film without montmorillonite with the corresponding film with the same lignin content and 3% of montmorillonite, in contrast to the 11.4% reduction in H-3M with respect to HPMC indicated above. This could be due to the exfoliation of montmorillonite causing a uniform dispersion of montmorillonite layers in the polymer matrix, increasing the tortuosity of the path. However, this effect also limits the increase in water contact angle when both lignin and montmorillonite were present (45.9 ± 2.9° for H film compared to 44.8 ± 3.3° for H-3M-1L, 46.2 ± 4.5° for H-3M-3L and 53.6 ± 4.8° for H-3M-10L films). This is probably due not only to the intercalation of lignin between montmorillonite layers, but also to the presence of montmorillonite in form of mostly individual rolled layers along with few individual unrolled layers, instead of the typical montmorillonite packages composed of several layers observed in H-3M film (Fig. 6a–c).

Oxygen barrier is one of the most important properties in food packaging, since it can extend the shelf life of fresh products.^8^ Therefore, oxygen permeability (OP) was evaluated in some of the most relevant bionanocomposite films to study the effect of lignin and montmorillonite addition. The evolution of the oxygen pressure in the chamber across the films was evaluated (Fig. SI.9 in ESI†) and oxygen permeability, diffusion coefficient and solubility were calculated (Table 4). It was observed that the incorporation of 3% of montmorillonite reduced the oxygen permeability in a 65.8%, due to the increase in tortuosity of the bionanocomposite. Moura *et al.*^9^ also reported a high improvement in oxygen barrier (reduction of 88.8%) due to the addition of 2.5% of montmorillonite to HPMC. In the same way, other authors reported similar improvements (20–80%) after incorporation of this layered clay to other biopolymers.^15,45,46^ When lignin was incorporated in a 1% content (H-3M-1L), no significant changes were observed compared to H-3M, conserving the good oxygen barrier properties. However, when lignin content increased to 3%, an increase in oxygen permeability was found due to an increase in the diffusion coefficient. This coefficient depends on both the free volume in the film and the polymer chain flexibility.^47^ That is, when increasing the packaging defects, gaps and other structural features, the diffusion coefficient increases. Thus, the presence of lignin aggregates in H-3M-3L film (black dots in FE-SEM images, Fig. 4) could reduce the packing of the film, causing this increase in the diffusion coefficient. Nevertheless, H-3M-3L films still presented better oxygen barrier properties than HPMC, in agreement with Tedeschi *et al.*^34^ who reported a reduction in oxygen permeability when incorporating different lignin content to xylan-cellulose films.

**Table 4:** Oxygen permeability (OP), diffusion coefficient (*D*) and solubility (*S*) of oxygen through the film samples (at 30 °C and 1 bar)

Sample	OP × 10^7^ (mL m m^−2^ d^−1^ Pa^−1^)	*D* × 10^8^ (cm^2^ s^−1^)	*S* × 10^3^ (cm^3^ cm^−3^ cm Hg^−1^)
HPMC	5.120	5.15	1.53
H-3M	1.750	1.83	1.48
H-3M-1L	2.074	1.69	1.87
H-3M-3L	2.851	3.47	1.27

Taking into account the above, the bionanocomposite film with better barrier properties was H-3M-1L, which presented very good oxygen permeability (2.07 × 10^−7^ mL m m^−2^ d^−1^ Pa^−1^) compared not only with HPMC films (with or without montmorillonite^9^ or Thai essential oils^7^) or other biopolymer films, such as poly(butylene adipate-co-terephthalate) (PBAT) (with and without nisin),^36^ but also with typical plastic films, such as low density polyethylene (LDPE), high density polyethylene (HDPE), polypropylene (PP) or polystyrene (PS)^36^ (Table 5). On the other hand, the incorporation of 3% montmorillonite and 1% lignin improves the water vapor barrier properties in a 43.8% compared to the HPMC film, resulting in WVP similar or lower than that reported for other HPMC films with montmorillonite,^9^ Thai essential oils^7^ or cypress seed extract,^6^ as well as some bioplastics, such as agar incorporated with lignin.^32,44^ However, this WVP is still higher than that reported for other bioplastics, such as PLA–lignin films^42^ or chitosan–lignin films,^43^ and much higher than that of typical plastic films, such as LDPE, HDPE, PP or PS^36^ (Table 5). Therefore, in applications requiring high water vapor barrier properties, other strategies such as multilayer systems could be studied.

**Table 5:** Oxygen permeability (OP) and water vapor permeability (WVP) of H-3M-1L compared to other biopolymers (PLA: polylactic acid; PBAT: poly(butylene adipate-*co*-terephthalate)); and plastics (LDPE: low density polyethylene; HDPE: high density polyethylene; PP: polypropylene; PS: polystyrene)

Sample	OP × 10^7^ (mL m m^−2^ d^−1^ Pa^−1^)	WVP × 10^11^ (g m m^−2^ s^−1^ Pa^−1^)	References
H-3M-1L	2.07	10.1	This work
HPMC with montmorillonite	1.82–0.06	8.1–5.8	9
HPMC with cypress seed extract	—	6.9–5.2	6
HPMC with Thai essential oils	6.38–4.54	90.0–65.2	7
Agar with lignin	—	211–153	32 and 44
PLA with lignin	—	2.9–2.4	42
Chitosan with lignin	—	0.18–0.15	43
PBAT	4.8	3.0	36
PBAT with nisin	11.3–7.54	3.61–3.4	36
LDPE	44.8	0.009–0.007	36
HDPE	7.1	0.003–0.002	36
PP	9.9–4.9	0.004–0.002	36
PS	14.8–9.9	0.046–0.011	36

### 3.7. Light barrier properties and color evaluation

**Fig. 11:**
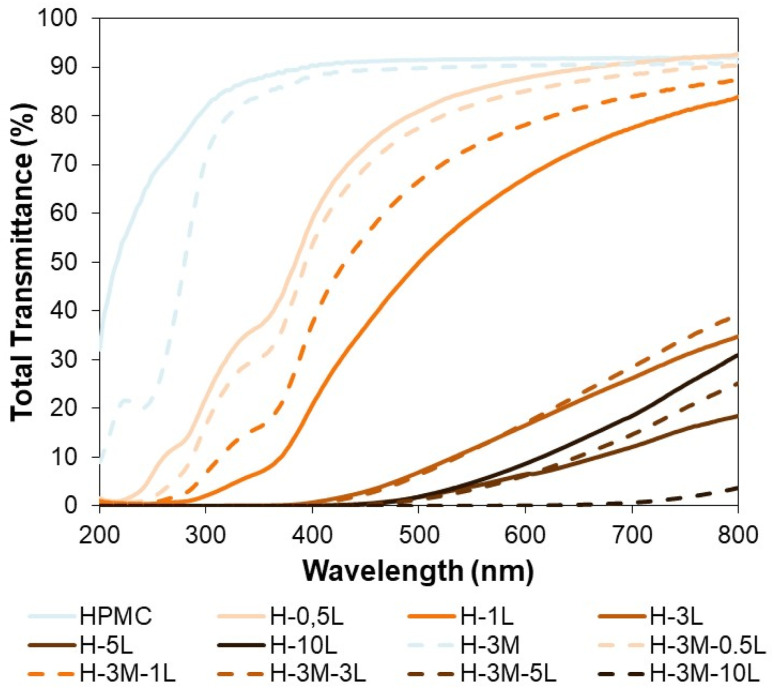
Total transmittance of the bionanocomposite films from 200 to 800 nm.

A good protection from UV radiation is another desirable feature in food packaging. HPMC films are transparent films with high transmittance in the visible region (400–700 nm) but also in the UV region (100–400 nm). Therefore, when UV-protection is required, the introduction of lignin in the bionanocomposite film could be a good option, as it has been proved for other biopolymers.^42,44,48,49^ This UV-shielding is attributed to the phenolic and conjugated carbonyl groups of lignin.^42,48,49^ Thus, the incorporation of only 0.5% lignin, reduced the transmittance at 315–280 nm (UV-B, the most energetic component of natural UV light) from 84.7–76.1% to 28.7–13.5% (Fig. 11). When the lignin content increased to 1%, the transmittance at 315–280 nm decreased to 3.5–0.9% achieving also a good blocking of UV-A (400–315 nm: transmittance of 20.8–3.5% *vs.* 90.4–84.7% for HPCM) while maintaining a good transmittance in the visible region (67.2% at 600 nm).

Furthermore, for lignin content of 3% or higher, a complete blocking of UV light was achieved, although the transmittance at the visible region also decreased significantly. On the other hand, the introduction of montmorillonite in the bionanocomposite films, caused some changes in transmittance, but did not have a significant effect on UV-shielding, except when lignin was not present, showing a reduction in transmittance at 315–280 nm from 84.7–76.1% (HPMC) to 78.4–22.9% (H-3M).

The color of the film samples could be also important in some packaging applications. Thus, color changes were evaluated based on CIE L*a*b* color coordinates (Table 6), where L* is related to the lightness of the sample and a* and b* coordinates are related to green-red and blue-yellow opponent colors, respectively. Due to the dark brown color of lignin, its incorporation into the HPMC matrix significantly reduced the lightness (L*) and increased b* coordinate (samples become more yellowish). However, a* coordinate was reduced for 0.5% lignin content and increased for lignin content higher than 3%, while no significant changes were found for 1% lignin content. Similarly, Rhimi *et al.*^6^ reported that increasing the concentration of cypress seed extract in HPMC film decreased lightness and increased both a* and b* coordinates, rendering samples more reddish and yellowish. Contrarily, the incorporation of montmorillonite did not cause significant color changes in either lignin-free (H *vs.* H-3M) or lignin-containing films (*i.e.* H-0.5L *vs.* H-3M-0.5L).

**Table 6:** CIE L*a*b* color coordinates of the bionanocomposite films and color changes (Δ*E*) compared to HPMC film

Sample	L*	a*	b*	Δ*E*
HPMC	95.28	−0.40	3.19	—
H-0.5L	89.04	−1.68	18.35	16.44
H-1L	82.44	−0.20	28.13	28.05
H-3L	64.86	5.81	27.78	39.61
H-5L	55.67	6.12	25.62	45.98
H-10L	45.07	4.30	23.43	54.34
H-3M	94.52	−0.47	3.63	0.88
H-3M-0.5L	88.78	−1.61	18.31	16.50
H-3M-1L	83.95	−0.55	27.36	26.69
H-3M-3L	71.25	7.36	30.66	37.31
H-3M-5L	53.12	7.33	29.32	50.20
H-3M-10L	45.21	4.89	23.77	54.39

### 3.8. Antioxidant capacity

**Fig. 12:**
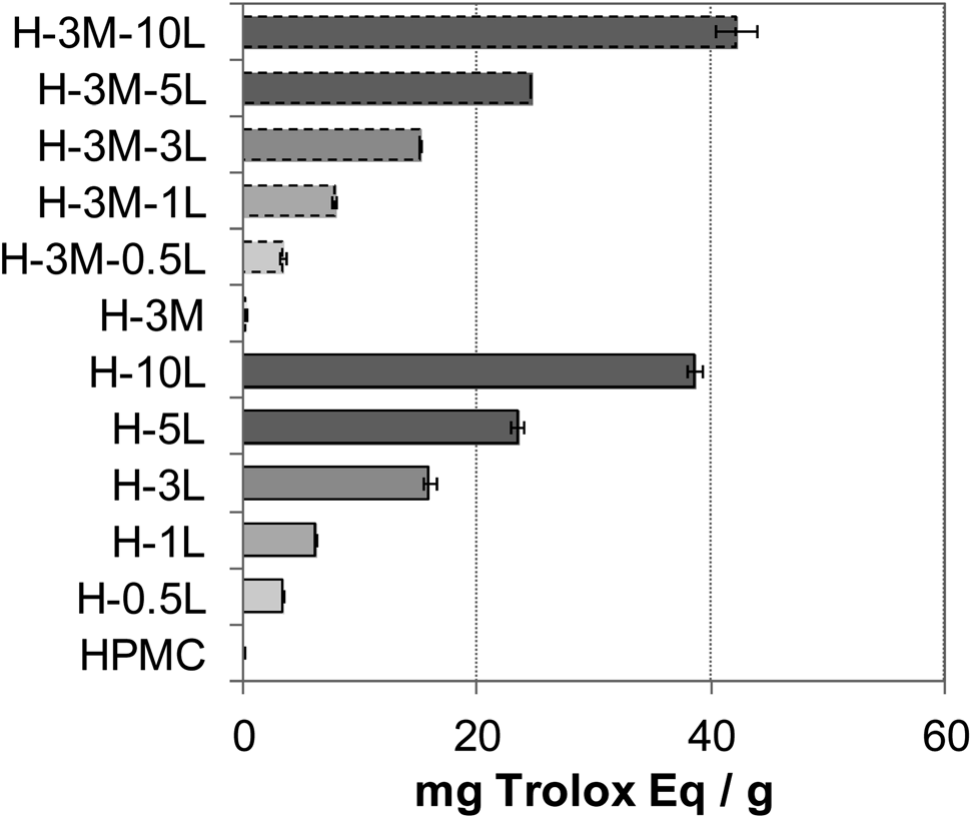
Antioxidant capacity of the bionanocomposite films, expressed as mg Trolox equivalent per gram of film.

Food packaging with antioxidant capacity can protect the food from oxidation which could lead to off-flavor, nutrient decomposition or toxic material production.^8^ The presence of phenolic groups in lignin, with free radical scavenging ability, confers antioxidant capacity to lignin-containing bionanocomposites.^2,50,51^ Thus, an increasing antioxidant capacity was found with increasing lignin content in the bionanocomposite films (Fig. 12), in agreement with results reported for other lignin-containing bionanocomposites.^34^ In fact, a linear relationship was found between lignin content and antioxidant capacity (Fig. SI.10, in ESI†). When comparing with other lignin-containing composite films previously studied, similar antioxidant capacity values were found. Thus, Gerbin *et al.*^52^ reported a percentage of inhibition using ABTS˙* method of 60–63% for films made of cellulose nanofibers (CNF) or cellulose nanocrystals (CNC) containing approx. 10% lignin, similar to that found for H-10L and H-3M-10L (54.2–60.5%).

### 3.9. Antibacterial activity

**Fig. 13:**
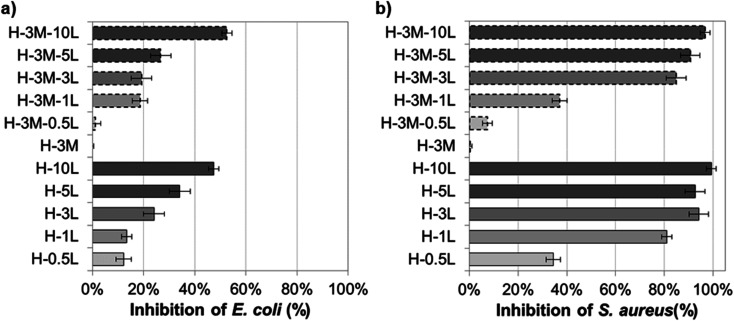
Antibacterial capacity of bionanocomposite films compared to HPMC film against (a) *E. coli* and (b) *S. aureus*.

Although HPMC film has no antimicrobial activity, preservatives and other antimicrobial agents can be incorporated conferring this property to the resulting bionanocomposite film. Thus, the microbial growth can be controlled prolonging the shelf life of fresh food when using these bionanocomposites as food packaging. Successful results have been obtained by incorporating plant-based extract such as propolis, essential oils (from oregano, bergamot, tea tree), nisin, preservatives (such as sodium benzoate and sodium propionate) or gentamicin–montmorillonite hybrid into HPMC.^7,8,16^ Lignin has proved also antimicrobial activity due to its phenolic groups and side chains with C_α_=C_β_, methyl group in the C_γ_ or functional groups such as methoxyl and epoxy groups, which can damage the cell membranes of microorganisms and cause lysis.^19,34,53–55^ Thus, when lignin was incorporated to the HPMC or H-3M films, growth inhibition of *E. coli* and *S. aureus* was found, not observing a clear effect of the presence or lack of montmorillonite (Fig. 13, SI.11 and SI.12†). For both bacteria, the higher the lignin content, the higher the inhibition, in agreement with other authors studying the addition of lignin in different polymer matrixes.^19^ The Gram-positive bacteria (*S. aureus*) showed higher inhibition than the Gram-negative bacteria (*E. coli*), probably due to the absence of membrane and the interaction of lignin with the dense peptidoglycan layer of Gram-positive bacteria.^19,38,56^ Nevertheless, other factors such as the ability of building exopolysaccharides, differences in the zeta potential, differences in the permeability of the outer membrane, different fatty acids, *etc.* could justify different level of antimicrobial activity depending on the bacteria.^19^ Similarly, Yang *et al.*^54^ reported higher antibacterial activity against *S. aureus* (99.2%) than against *E. coli* (96.5%) when 3% lignin nanoparticles were incorporated to polyvinyl alcohol (PVA) films. Gerbin *et al.*,^52^ who study CNF and CNC films with 10–17% lignin, also reported significant antibacterial activity against *S. aureus* (log *R* of 0.2–0.5, corresponding to growth inhibition of approx. 40–70%) while no bacterial viability reduction was clear against *E. coli*.

## 4. Conclusion

Bionanocomposite films based on HPMC, kraft lignin and montmorillonite showed improved properties. On the one side, the addition of montmorillonite significantly improved gas barrier properties, reducing both water vapor and oxygen permeability in 11% and 66%, respectively. Furthermore, a synergistic effect on improving water vapor permeability was observed when incorporating both lignin and montmorillonite to the HPMC film, probably due to montmorillonite exfoliation mediated by lignin, achieving a very good dispersion of the clay and lignin into the HPMC matrix. On the other side, the addition of lignin also conferred the HPMC bionanocomposite films with UV-shielding, antioxidant and antibacterial properties. Thus, the H-1L-3M film, with 1% lignin and 3% montmorillonite contents, kept the good mechanical and thermal properties of HPMC films, but presenting much better water vapor and oxygen permeability (decreases of 44% and 60%, respectively) and showing UV-protection (while maintaining good transparency) and antioxidant and antibacterial activity against *E. coli* and *S. aureus*. Taking into account all these remarkable characteristics, the resulting materials seem promising for application as active food packaging, with still room for optimization in future work by varying the content in montmorillonite. Moreover, the potential showed by kraft lignin for improving both exfoliation and dispersion of montmorillonite, highlighted its role in enhancing the compatibility between clays and polymeric matrixes, which could be of great interest in the development of a new type of bio-organo-clays.

## Conflicts of interest

There are no conflicts to declare.

## Supplementary Material

NA-005-D3NA00283G-s001
