# Supplementary material for: Effect of the combined addition of ultrasonicated kraft lignin and montmorillonite on hydroxypropyl methylcellulose bionanocomposites
Source: Nanoscale Adv. 2023 Jul 10;5(16):4107–23. doi: 10.1039/d3na00283g (PMC10408596; doi:10.1039/d3na00283g)
Supplement: NA-005-D3NA00283G-s001 [file NA-005-D3NA00283G-s001.pdf]

Electronic Supplementary Material (ESI) for Nanoscale Advances.
This journal is © The Royal Society of Chemistry 2023

SUPPORTING INFORMATION FOR:

Effect of the combined addition of ultrasonicated kraft lignin and montmorillonite on hydroxypropyl methylcellulose bionanocomposites

Raquel Martín-Sampedro[1,2,3], Pilar Aranda[1,3], Gustavo del Real[2], Eduardo Ruiz-Hitzky[1,3], Margarita Darder[1,3]*

[1] Materials Science Institute of Madrid (ICMM), CSIC, C/ Sor Juana Inés de la Cruz 3, 28049, Madrid, Spain

[2] National Institute of Agricultural and Food Research and Technology (INIA), CSIC, Ctra. de la Coruña, km 7.5. 28040, Madrid, Spain

[3]Interdisciplinary Platform for Sustainable Plastics towards a Circular Economy-Spanish National Research Council (SusPlast-CSIC), Madrid, Spain.

*raquel.martin@inia.csic.es

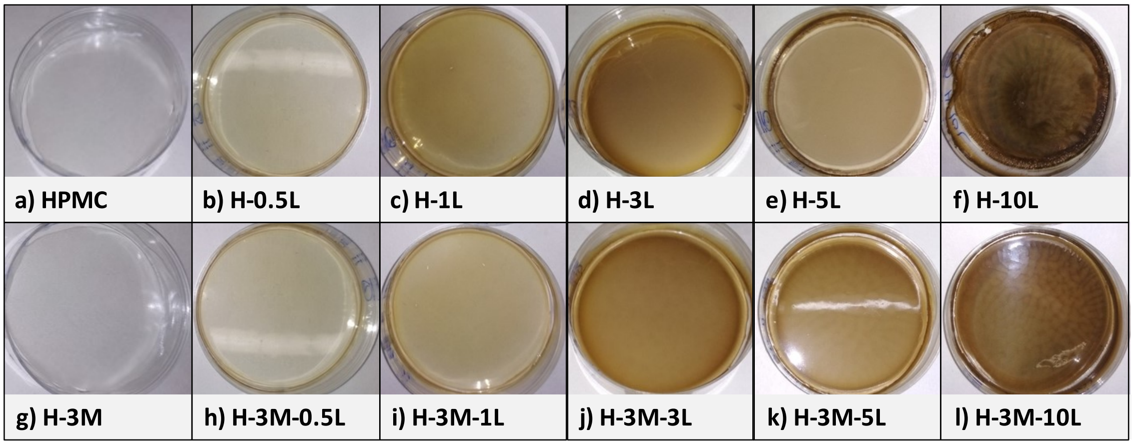

Figure SI.1. HPMC films without (a-f) and with (g-l) 3% montmorillonite and with different lignin content: 0% (a and g), 0.5% (b and h), 1% (c and i), 3% (d and j), 5% (e and k) and 10% (f and l).

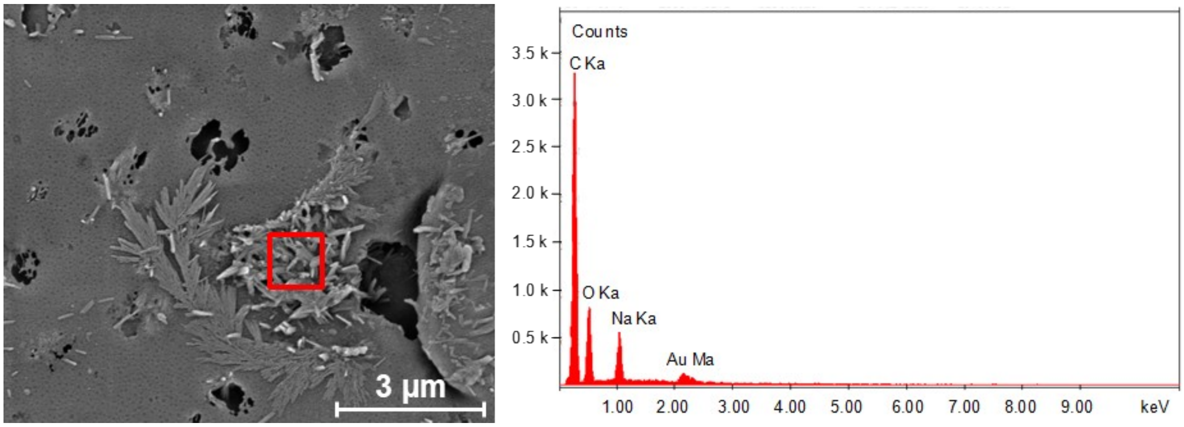

Figure SI.2. EDX analysis of staked rods observed in H-5L film (marked with a red square)

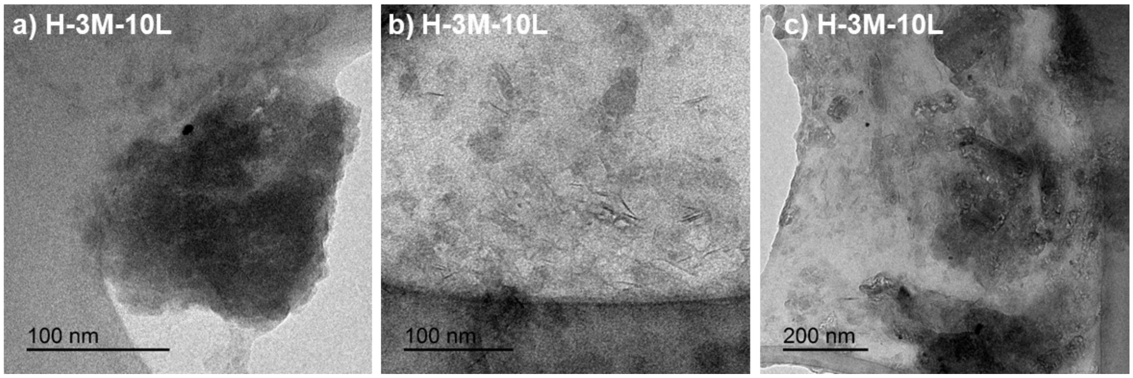

Figure SI.3. TEM images of HPMC films with 3% montmorillonite and 10% lignin.

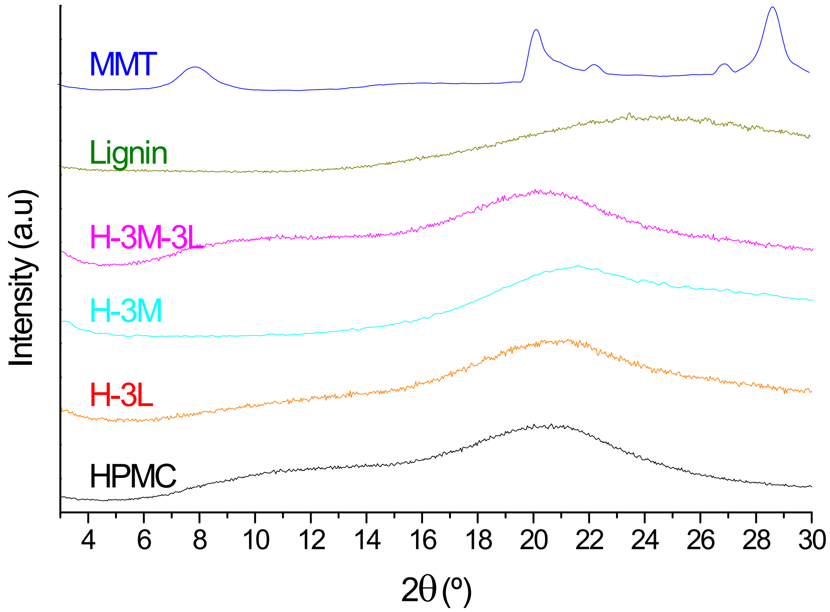

Figure SI.4. XRD patterns of montmorillonite (MMT), lignin and HPMC, H-3L, H-3M and H-3M-3L films.

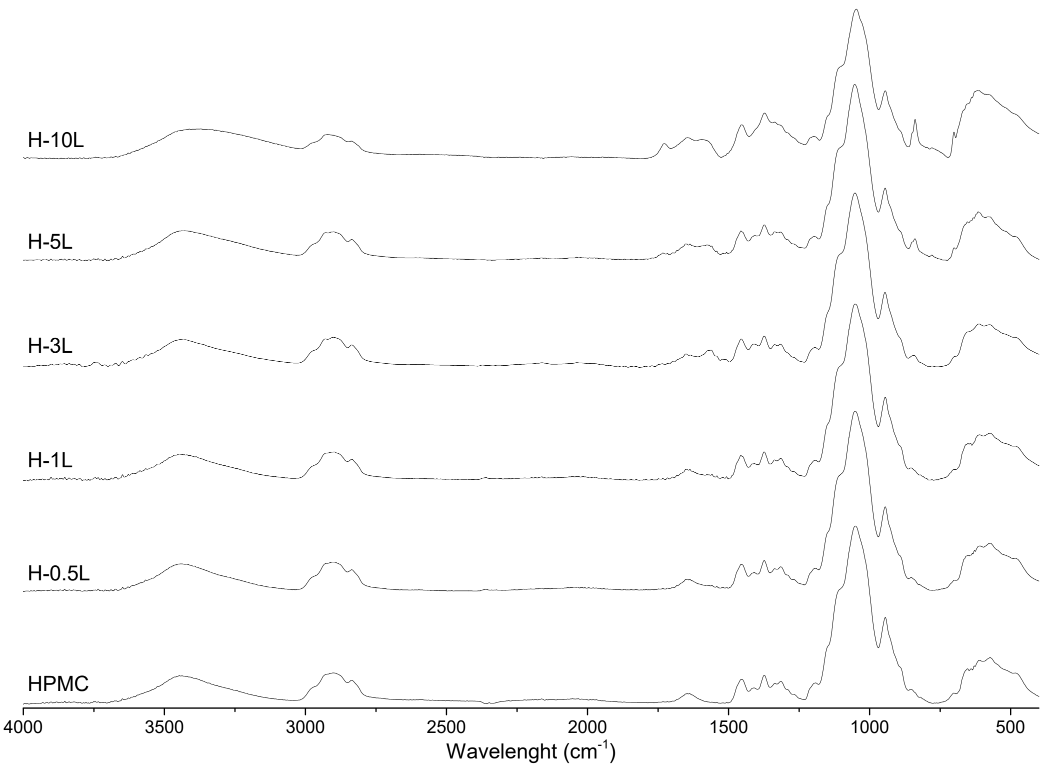

Figure SI.5. Fourier Transform Infrared (FTIR) spectra of HPMC without montmorillonite and with different lignin content (0-10%).

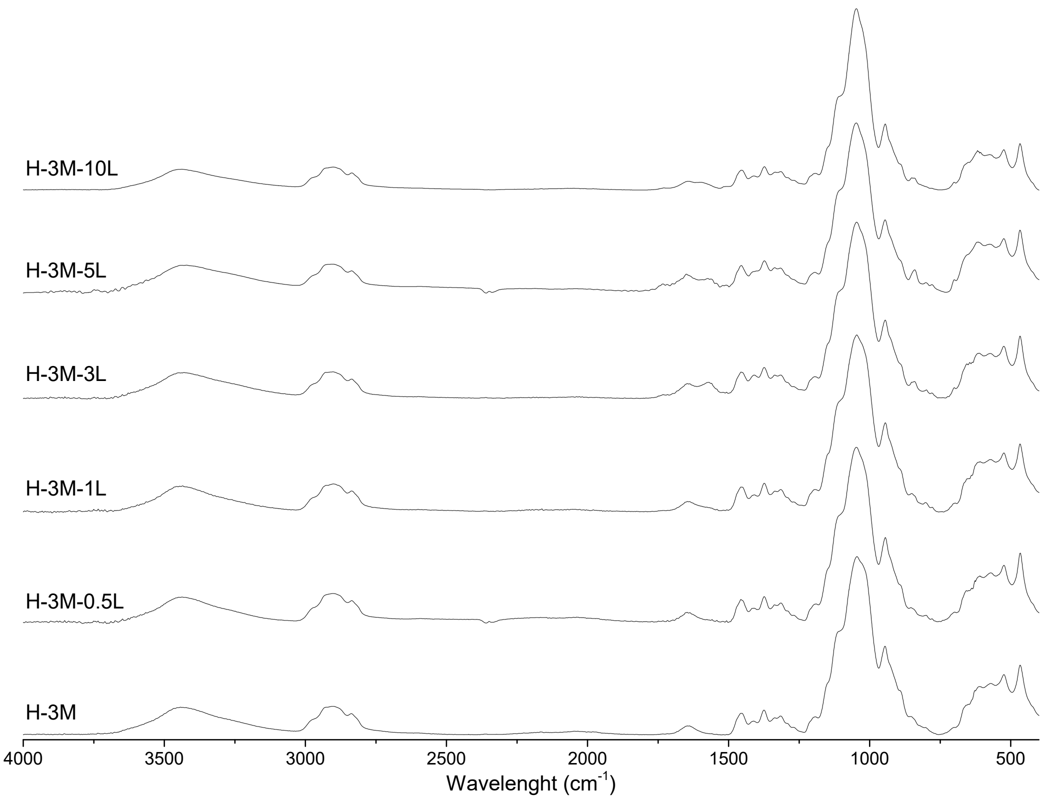

Figure SI.6. Fourier Transform Infrared (FTIR) spectra of HPMC film with 3% montmorillonite and with different lignin content (0-10%).

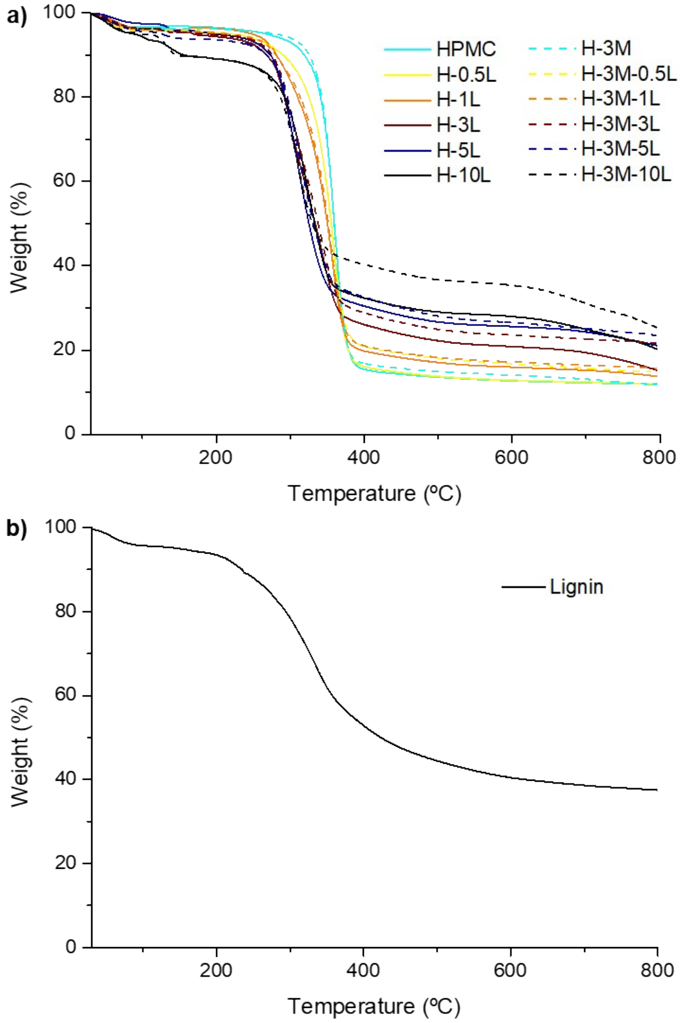

Figure SI.7. Thermogravimetric curves of a) the HPMC films without (continuous lines) and with montmorillonite (dash lines) and with different lignin content, and b) kraft lignin.

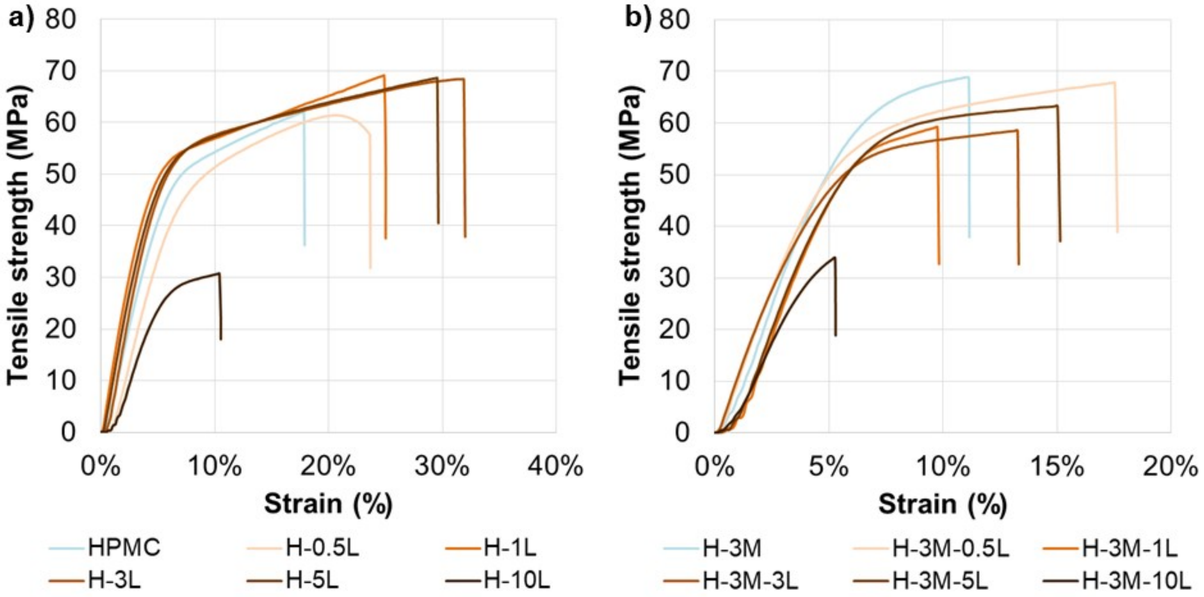

Figure SI.8. Stress-strain curves for bionanocomposite films a) without montmorillonite and b) with montmorillonite

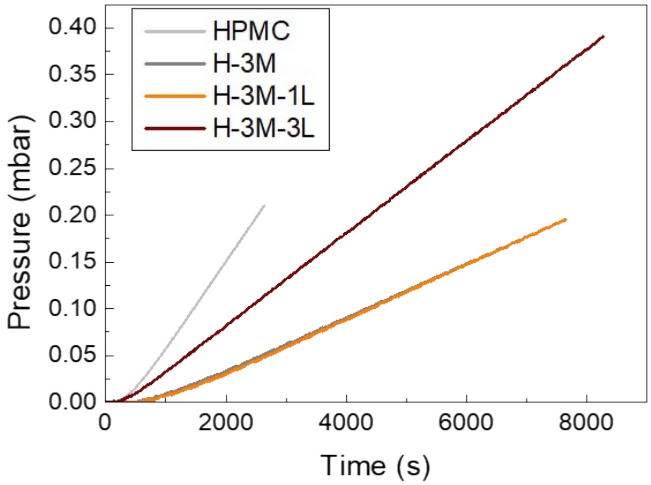

Figure SI.9. Evolution of the oxygen pressure in the low pressure chamber at 30ºC across HPMC, H-3M, H-3M-1L and H-3M-3L samples.

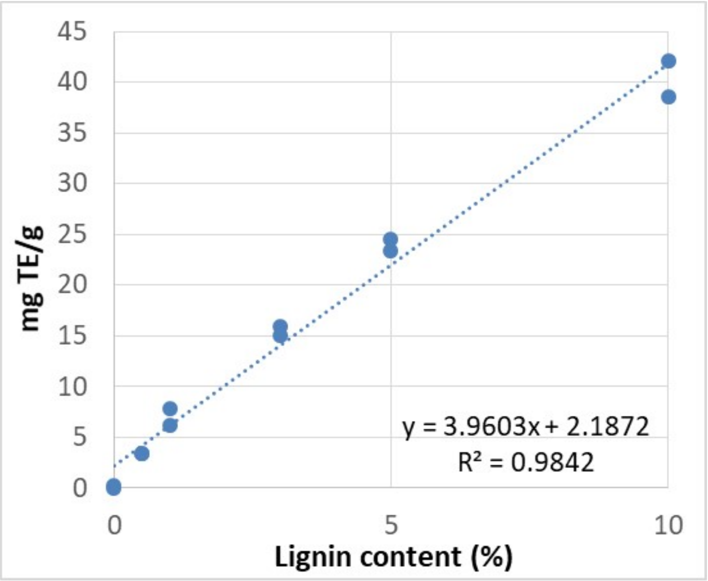

Figure SI.10. Linear relationship between antioxidant capacity and lignin content of the bionanocomposite films.

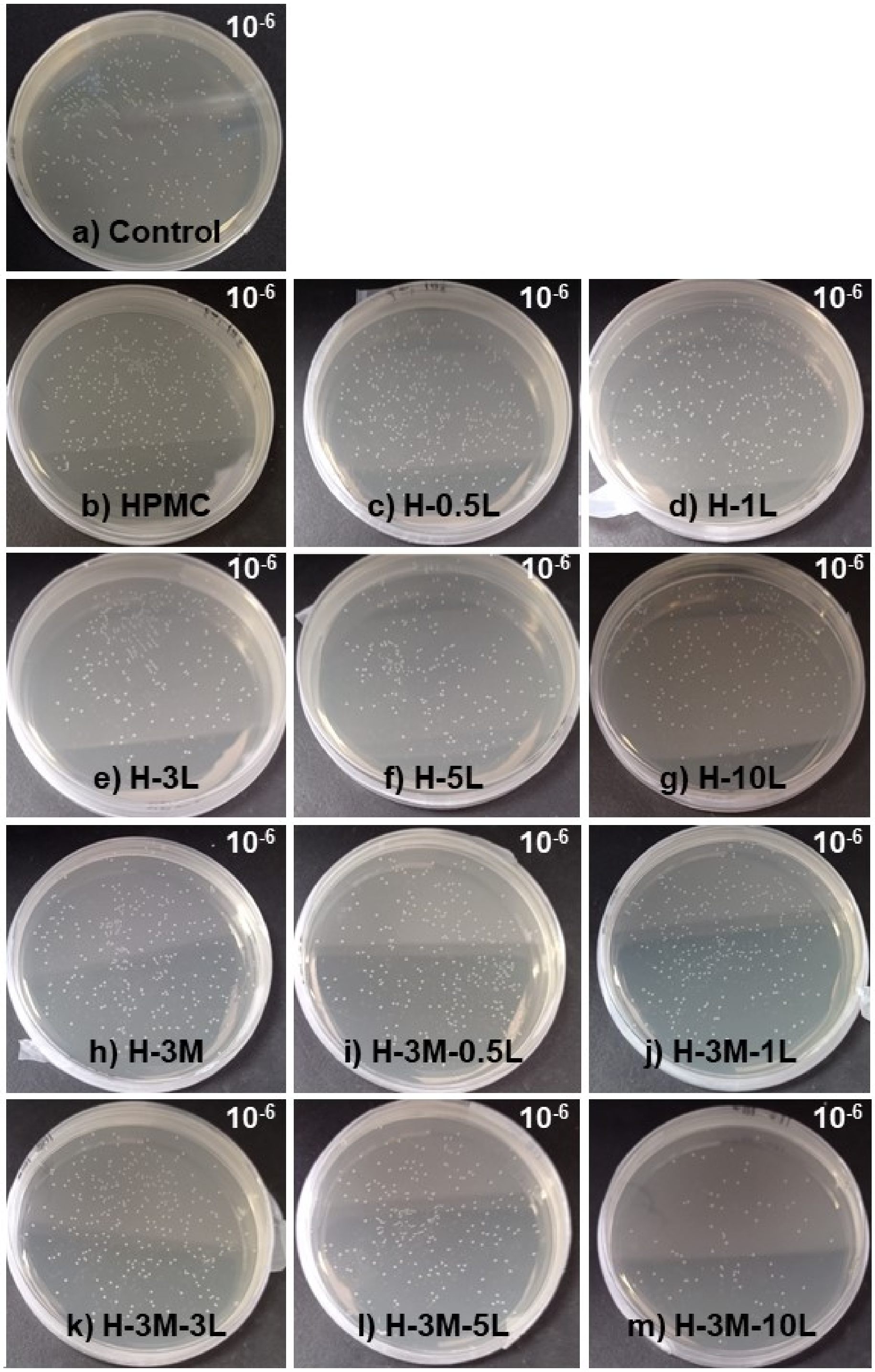

Figure SI.11. Photographs of the agar plates used for counting the number of *E. coli* colonies grown in the presence of each bionanocomposite film. Control sample without any film was also included, and indicated as control. All images correspond to 10^{-5} dilutions of the corresponding cultures.

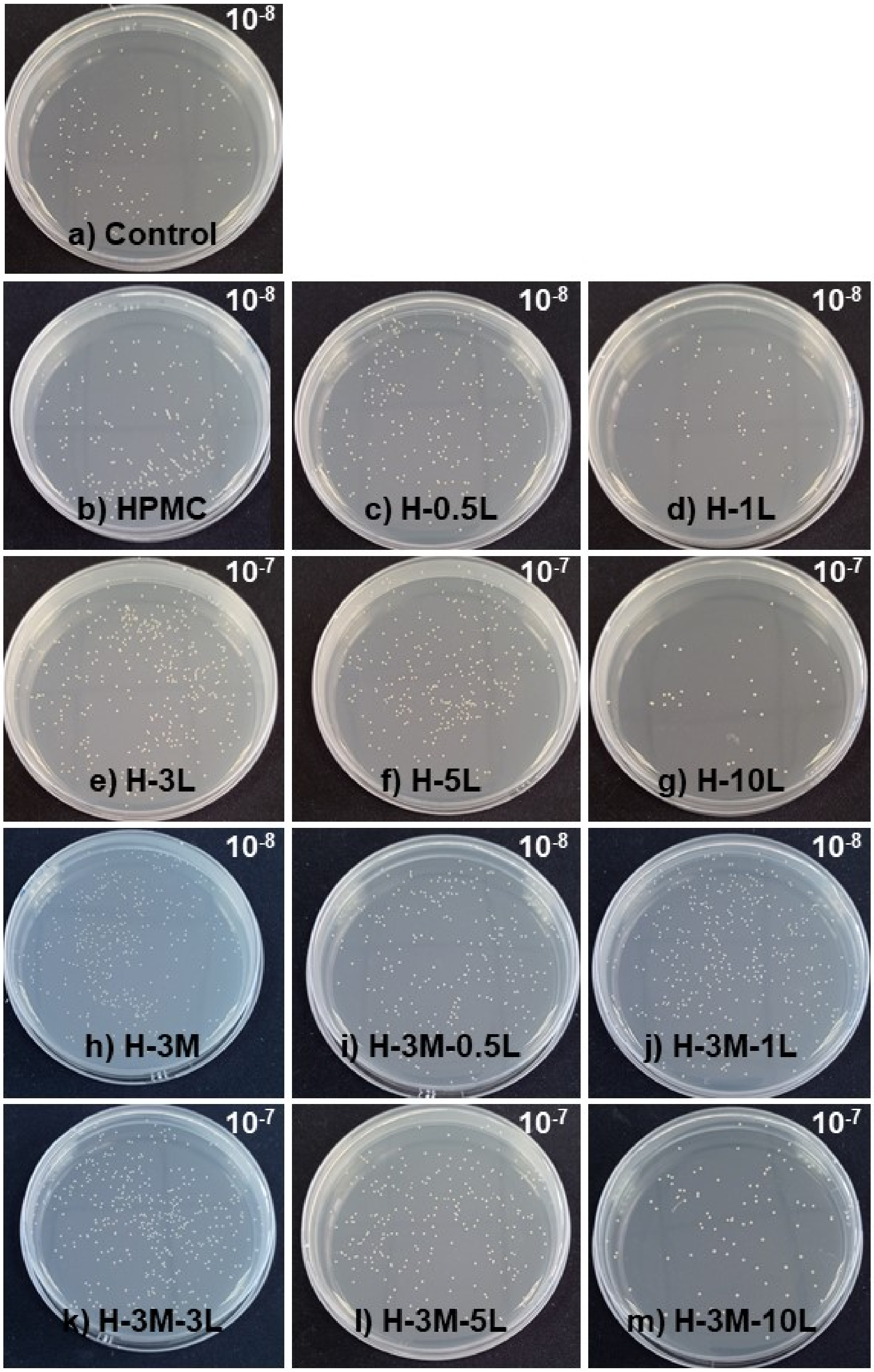

Figure SI.12. Photographs of the agar plates used for counting the number of *S. aureus* colonies grown in the presence of each bionanocomposite film. Control sample without any film was also included, and indicated as control. Images a-d and h-j correspond to 10^{-8} dilutions, and e-g and k-m to 10^{-7} dilutions of the corresponding cultures.
